# Supplementary material for: 4D structural biology–quantitative dynamics in the eukaryotic RNA exosome complex
Source: Nat Commun. 2025 Aug 24;16:7896. doi: 10.1038/s41467-025-62982-6 (PMC12375074; doi:10.1038/s41467-025-62982-6)

SUPPLEMENTARY MATERIAL

4D structural biology – Quantitative dynamics in the eukaryotic RNA exosome complex

Jobst Liebau[1*], Daniela Lazzaretti[1*], Torben Fürtges[2,3], Anna Bichler[1], Michael Pilsl[4], Till Rudack[2,3*] and Remco Sprangers[1]*

Affiliations

[1] Department of Biophysics I, Regensburg Center for Biochemistry, University of Regensburg, Universitätsstraße 31, 93053 Regensburg, Germany.

[2] Structural Bioinformatics Group, Regensburg Center for Biochemistry, University of Regensburg, Universitätsstraße 31, 93053 Regensburg, Germany.

[3] Structural Bioinformatics Group, Regensburg Center for Ultrafast Nanoscopy, University of Regensburg, Universitätsstraße 31, 93053 Regensburg, Germany.

[4] Structural Biochemistry Group, Regensburg Center for Biochemistry, University of Regensburg, Universitätsstraße 31, 93053 Regensburg, Germany.

* Corresponding Authors: Remco Sprangers (general remarks), E-mail: remco.sprangers@ur.de, phone: +49-941-943-7751, ORCID: 0000-0001-7323-6047, specific remarks can also be addressed to: Jobst Liebau (NMR), E-mail: jobst.liebau@ur.de, Till Rudack (MD), E-mail: till.rudack@ur.de, Daniela Lazzaretti (X-ray and cryo-EM structures), E-mail: daniela.lazzaretti@ur.de

Table of contents

Supplementary Figures S1-S28	Page S2
Supplementary Tables S1-S10	Page S37
Supplementary References	Page S58
Uncropped gels	Page S59

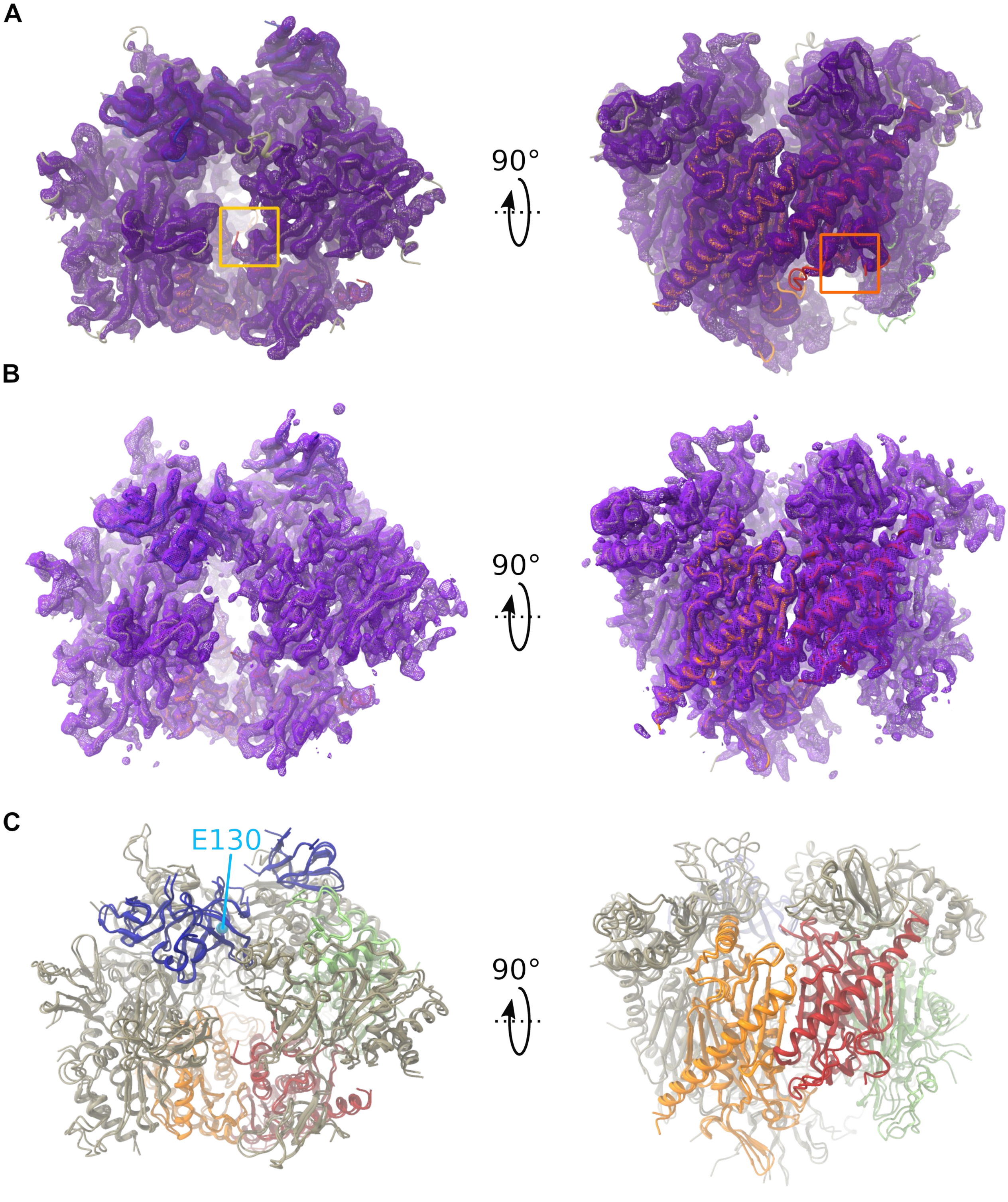
A
90°
B
90°
C
E130
90°

D

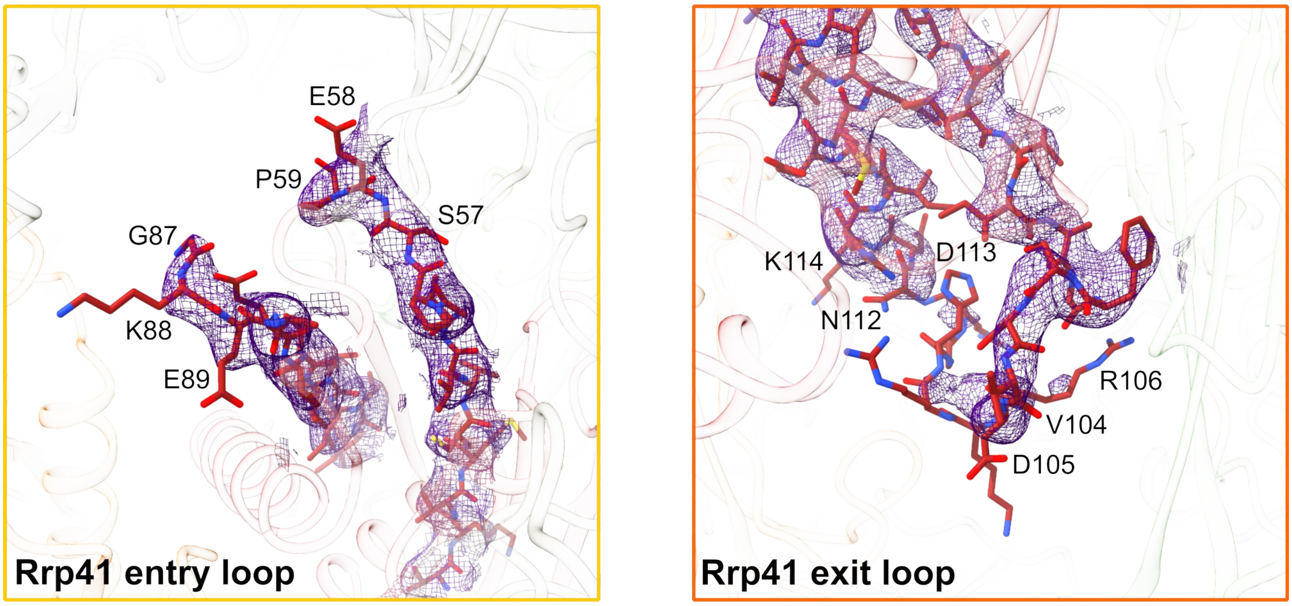

E

ctRrp41 51 CVVTGPSEP**GPRRGTGAGTTGGGGAGGAGGGGSGGQ**GKEAEVV 93
cryo-EM SSSS----------------------------------SSS

ctRrp41 94 VSIVIAGFSSVDR**KRHGR**NDKRII 117
cryo-EM SSSSS-------------HHHHH

ctRrp42 70 VKAEVEKT**VSRSKEDEEVGLLVASAGDMDVDDEEGYAKVGADNR**TGEASWVEITVE 125
cryo-EM SSSSSSS---SSSSS

Figure S1: Structure of the *C. thermophilum* exosome core ctExo9. (**A**) Top view (left) and side view (right) of the cryo-EM density map at 3.2 rmsd. The close-ups in panel **D** are located inside the colored rectangles. (**B**) Top view (left) and side view (right) of the X-ray crystallography electron density map at 1 rmsd. (**C**) Overlay of the cryo-EM and X-ray crystallography structures. Csl4 is in blue, Rrp41 is in red, Rrp45 is in orange and Rrp42 is in green. Csl4 E130 is highlighted. (**D**) Cryo-EM density around the invisible section of the Rrp41 entry loop (left, yellow rectangle in panel **A**) and around the invisible section of the Rrp41 exit loop (right, orange rectangle in panel **A**), contoured at 3.1 rmsd. (**E**) Sequence and secondary structure obtained from the cryo-EM structure around the entry loop of Rrp41 (top), the exit loop of Rrp41 (center) and Rrp42-EL (bottom). S = strand, H = helix, - = loop or invisible residue. Residues in bold are invisible in the cryo-EM structure.

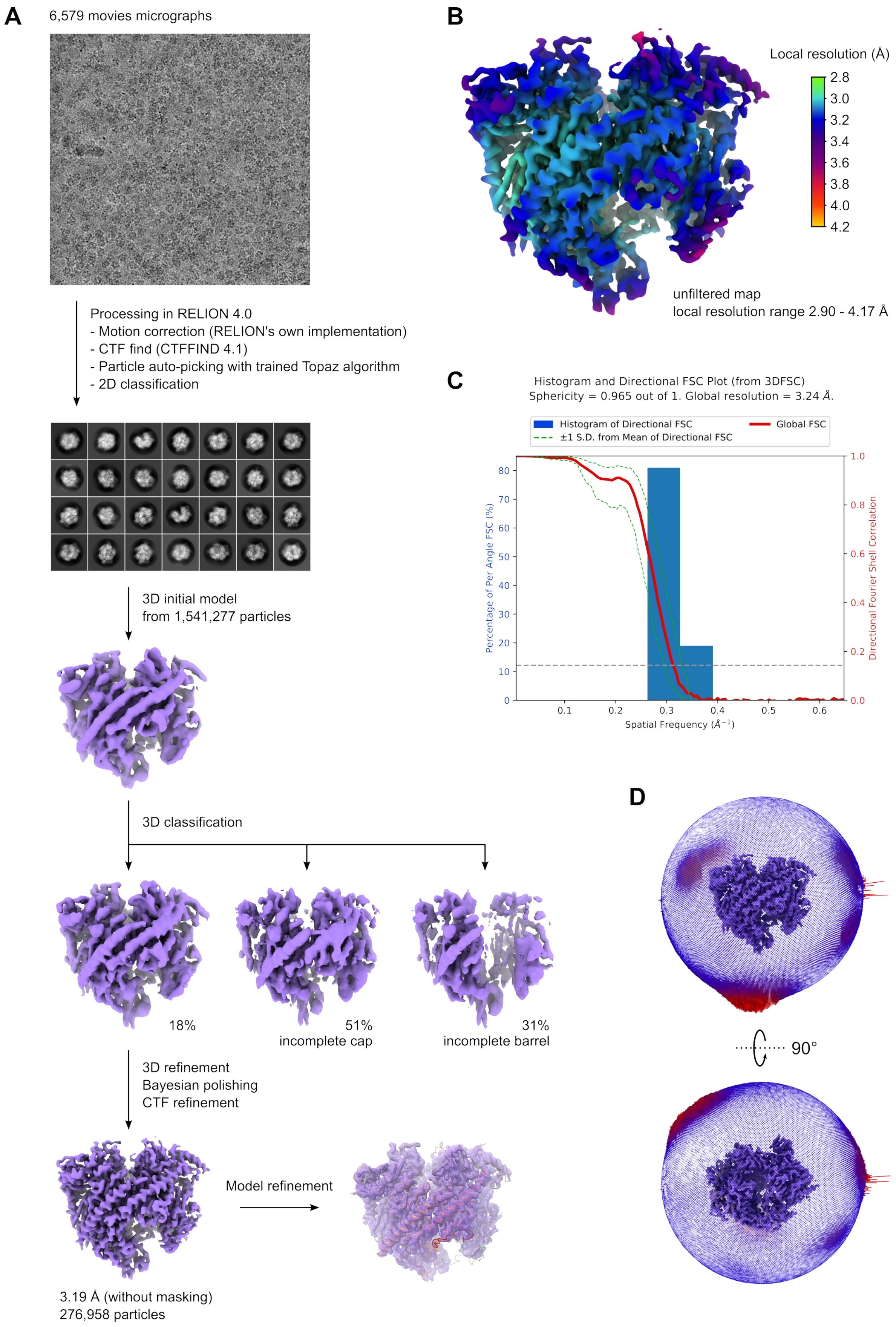
A
6,579 movies micrographs
Processing in RELION 4.0
- Motion correction (RELION's own implementation)
- CTF find (CTFFIND 4.1)
- Particle auto-picking with trained Topaz algorithm
- 2D classification
3D initial model
from 1,541,277 particles
3D classification
18%
51%
incomplete cap
31%
incomplete barrel
3D refinement
Bayesian polishing
CTF refinement
Model refinement
3.19 Å (without masking)
276,958 particles
B
Local resolution (Å)
2.8
3.0
3.2
3.4
3.6
3.8
4.0
4.2
unfiltered map
local resolution range 2.90 - 4.17 Å
C
Histogram and Directional FSC Plot (from 3DFSC)
Sphericity = 0.965 out of 1. Global resolution = 3.24 Å.
Histogram of Directional FSC
±1 S.D. from Mean of Directional FSC
Global FSC
Percentage of Per Angle FSC (%)
Directional Fourier Shell Correlation
Spatial Frequency (Å⁻¹)
D
90°

Figure S2: Cryo-EM data processing. (**A**) Scheme of the cryo-EM processing pipeline. The raw final density map of ctExo9, reconstructed from 276,958 particles, is shown at the bottom. The global resolution is 3.19 Å at FSC = 0.143. (**B**) Final Exo9 density map (as in **A**, bottom), contoured at 4 rmsd and colored according to the local resolution (calculated in RELION). (**C**) 3DFSC plot (1) calculated from the raw, unfiltered half maps. The sphericity of 0.965 indicates a very isotropic angular distribution. (**D**) Angular distribution of the particles used for the final reconstruction, plotted on the final map shown in panel **A**, bottom.

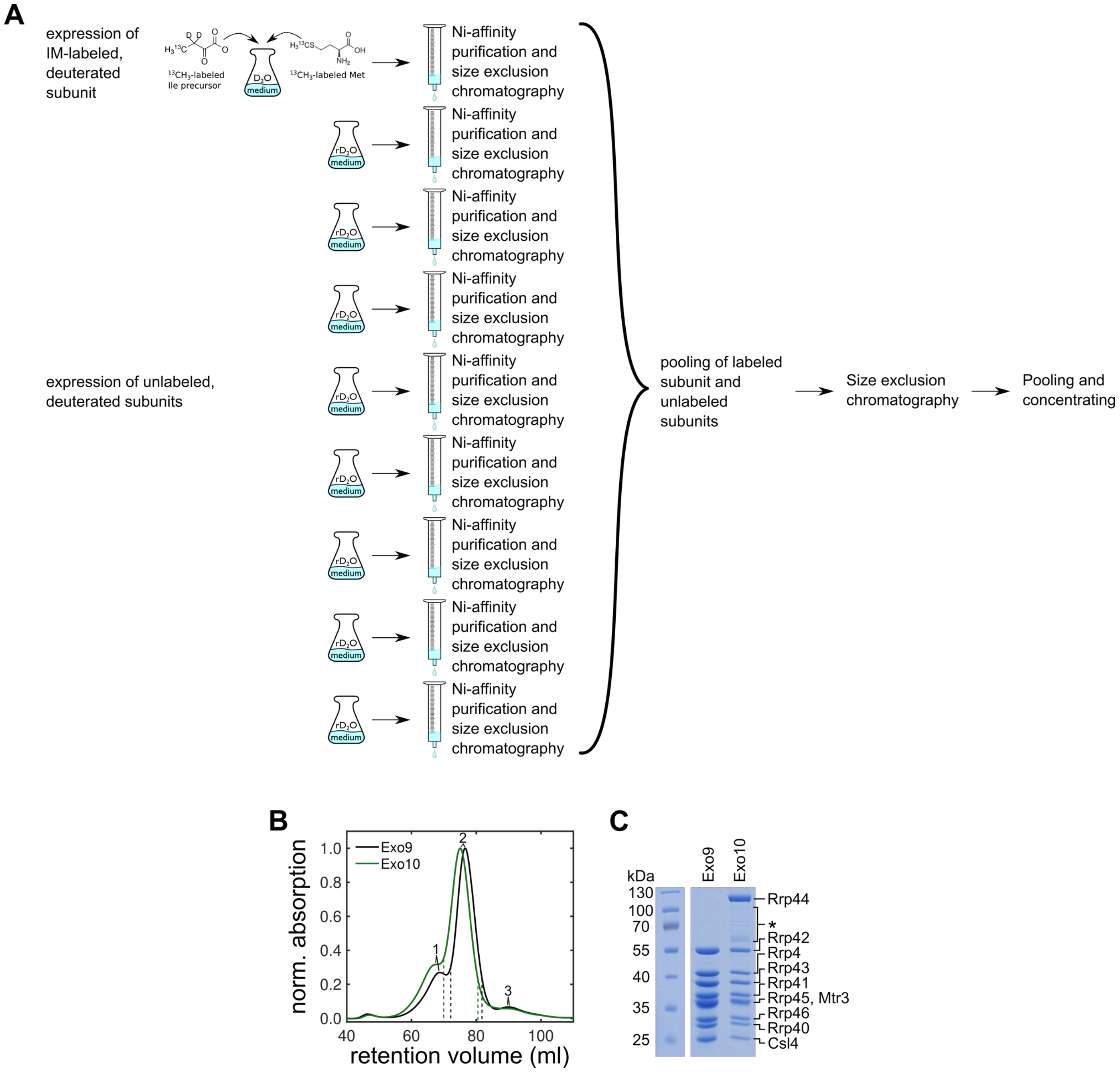

Figure S3: Reconstitution of Exo9 and Exo10 complexes from individual subunits. (**A**) Sample preparation to obtain modularly labeled exosome complexes (this example is for IM-labeling; reconstitutions of ^{19}F or TEMPO spin-labeled subunits were performed analogously). The labeled subunit is expressed in D_2O medium in the presence of appropriate precursors and purified. The unlabeled subunits are expressed individually (or as heterodimers) in ~95% D_2O (rD_2O) medium and purified. The labeled subunit is pooled with the (8 or 9 other) unlabeled subunits followed by a size-exclusion purification step. Peak fractions are pooled and concentrated. (**B**) Size exclusion chromatogram of the exosome complex reconstituted *in vitro*. Exo9 is in black and Exo10 is in green. The absorption was measured at 280 nm and is normalized to the maximum absorption of each trace. Fractions between the dashed green/black lines were pooled. Elution peak 1 corresponds to exosome aggregates, isolated exosome complexes give rise to elution peak 2 and excess subunits

that did not form complexes give rise to elution peak 3. (**C**) SDS-PAGE gel of the pooled peak fractions of Exo9 and Exo10. Subunits are labeled. * denotes impurities that arise from the purification of Rrp44. Note that the concentration of Exo9 is ca. 2x the concentration of Exo10 explaining stronger bands for the Exo9 gel. The gels suggest stoichiometric reconstitution of the complex and are analogous to previously published gels of *S. cerevisiae* exosome complexes (2).

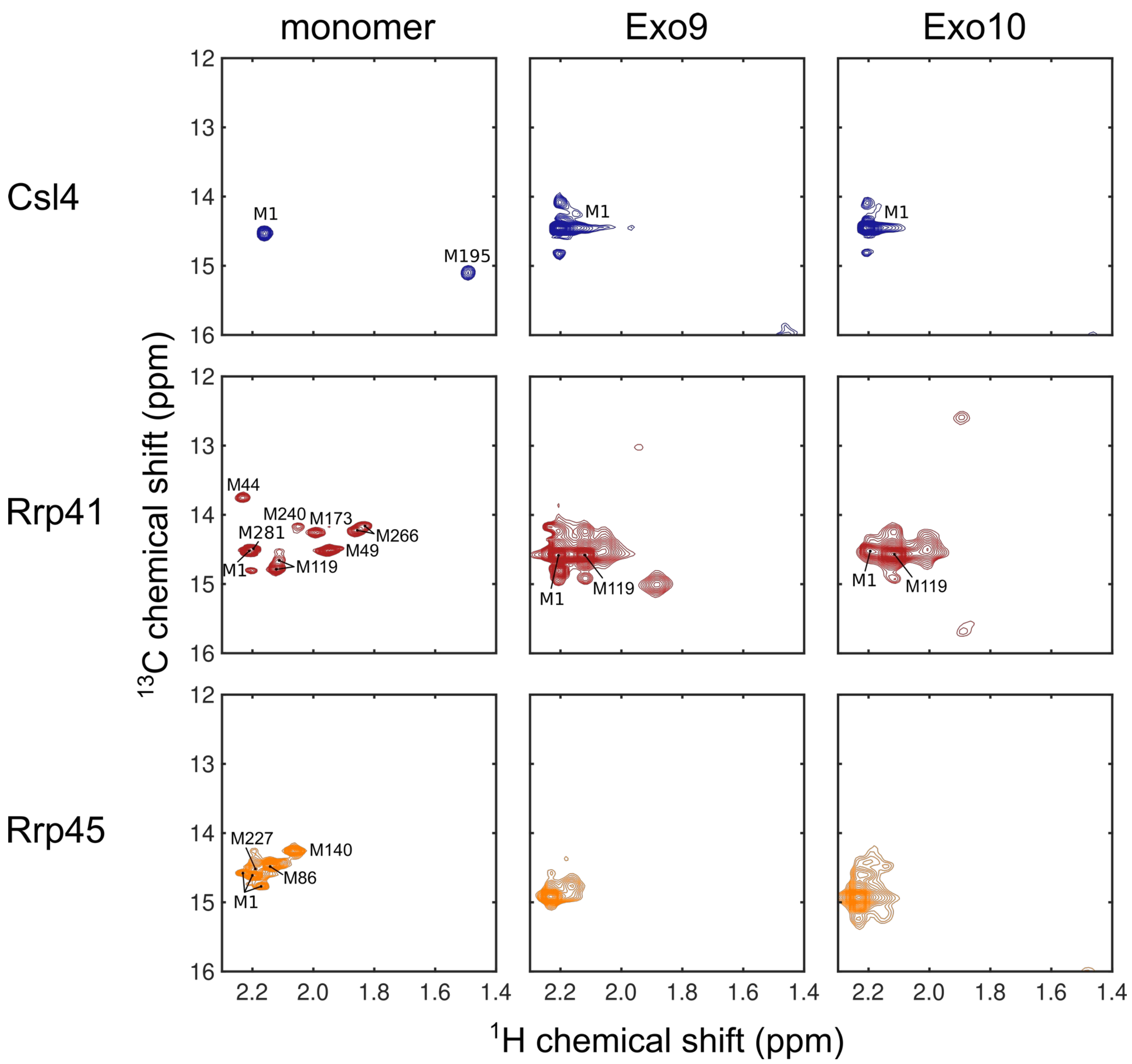

Figure S4: Met-ε1 methyl-TROSY spectra. Met-ε1 region of methyl-TROSY spectra for Csl4 (blue), Rrp41 (red) and Rrp45 (orange) as monomers (column 1) and when reconstituted into Exo9 (column 2) and Exo10 (column 3). Note, that the methionine resonances in the Exo9 and Exo10 complexes suffered from significant spectral overlap and were not suitable for the extraction of quantitative NMR parameters.

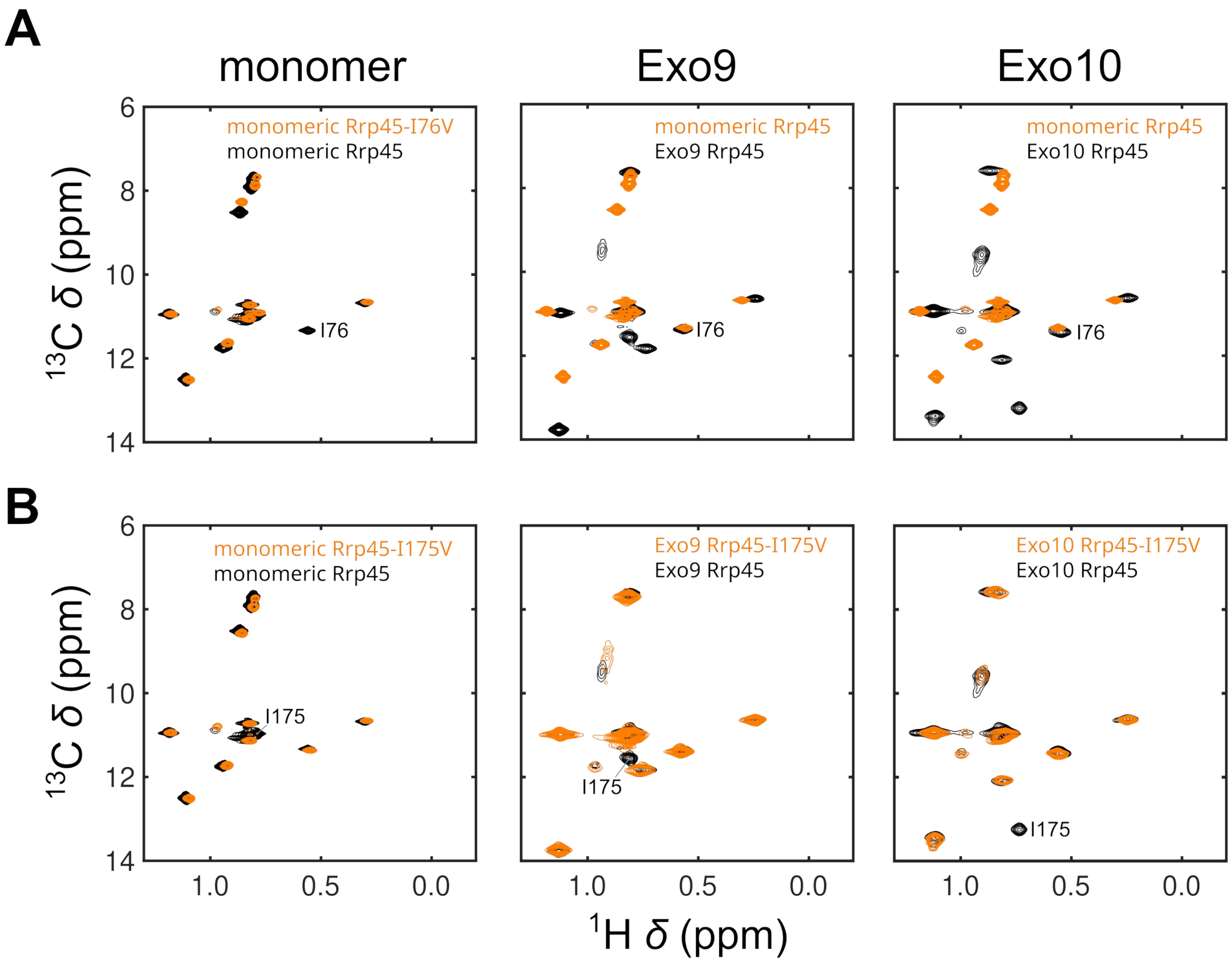

Figure S5: Examples of assignment by point mutation. (**A**) Assignment of a resonance (I76) that does not change chemical shift significantly between the monomeric form and the Exo9 or Exo10 complexes. Residue I76 of Rrp45 is assigned by comparing the methyl-TROSY spectrum of IM-labeled monomeric Rrp45 (left panel, black) to the spectrum of IM-labeled monomeric Rrp45-I76V (left panel, orange). Since the peak position of I76 is not strongly affected by reconstitution into Exo9 or Exo10, the assignment of monomeric Rrp45-I76 can be transferred to IM-labeled Rrp45 reconstituted into Exo9 (central panel, black) and Exo10 (right panel, black). IM-labeled monomeric Rrp45 is shown in orange in the central and right panel. (**B**) Assignment of a resonance that changes its chemical shift significantly upon formation of the Exo9 or Exo10 complexes. Residue I175 of Rrp45 is assigned by comparing the methyl-TROSY spectrum of IM-labeled monomeric Rrp45 (left panel, black) to the spectrum of IM-labeled monomeric Rrp45-I175V (left panel, orange). Since the peak position of I175 shifts substantially upon reconstitution into Exo9 (central panel, black) or Exo10 (right panel, black), the assignment in Exo9 and Exo10 is obtained by reconstituting IM-labeled Rrp45-I175V into Exo9 (central panel, orange) and Exo10 (right panel, orange).

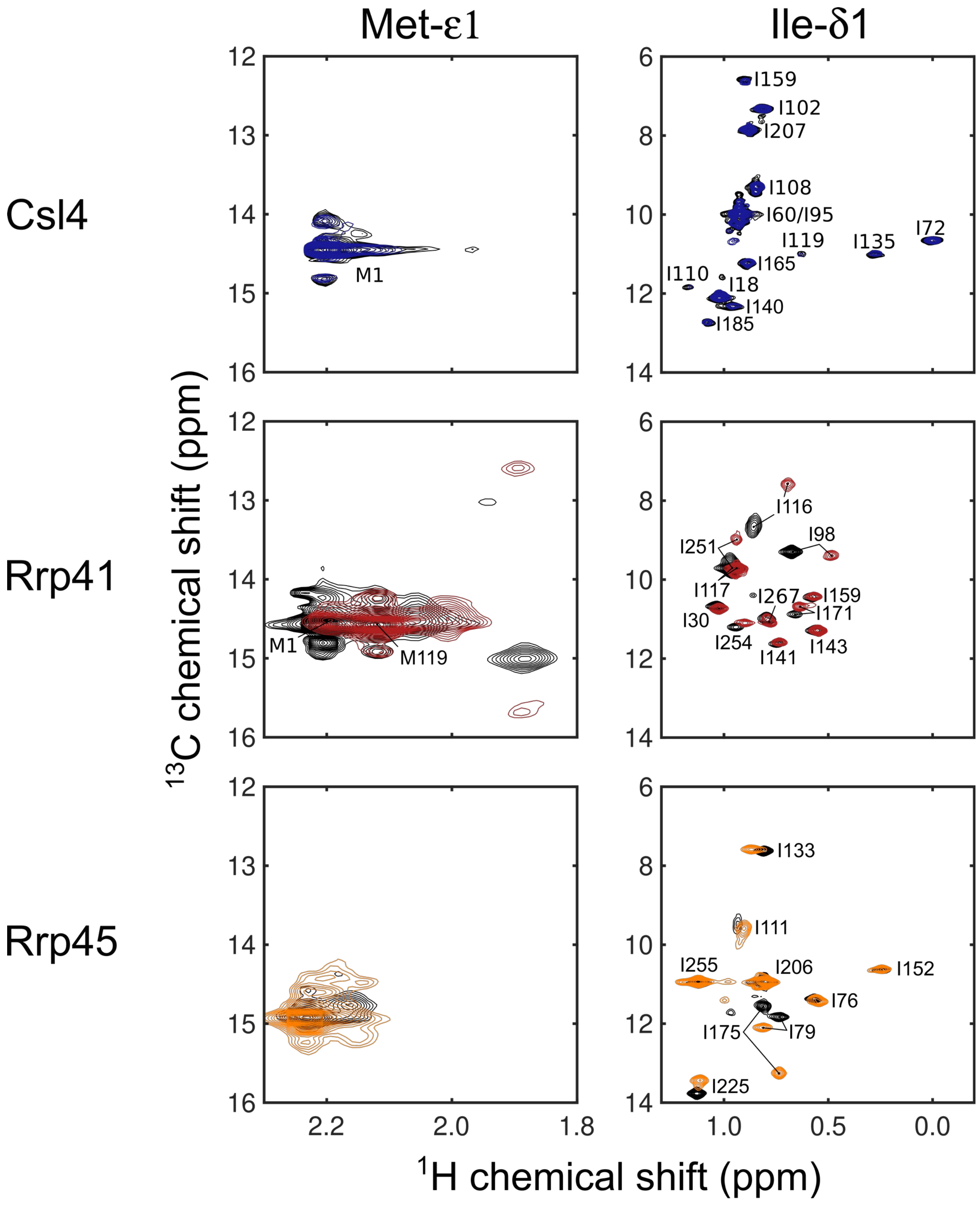

Figure S6: Interaction between Exo9 subunits and Rrp44. Overlay of the Met-ε1 (left) and Ile-δ1 (right) regions of methyl-TROSY spectra of IM-labeled Csl4 (blue), Rrp41 (red) and Rrp45 (orange) reconstituted into Exo9 (black) and Exo10 (in color).

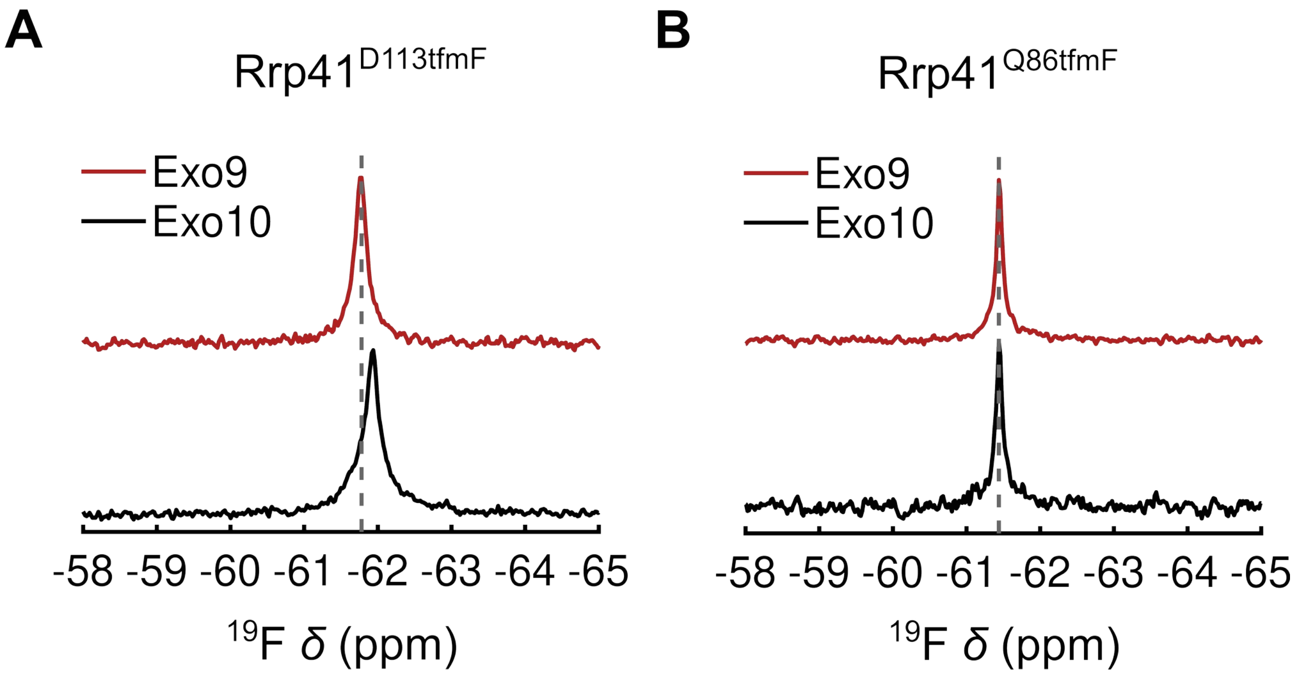

Figure S7: Interaction of the exit and entry loop of Rrp41 with Rrp44. (**A**) 1D ^{19}F spectrum of Rrp41^{D113tfmF} (exit loop) and (**B**) 1D ^{19}F spectrum of Rrp41^{Q86tfmF} (entry loop) reconstituted into Exo9 (red) and Exo10 (black). The dashed line indicates the center of the resonance for Exo9. The exit loop senses the presence of Rrp44.

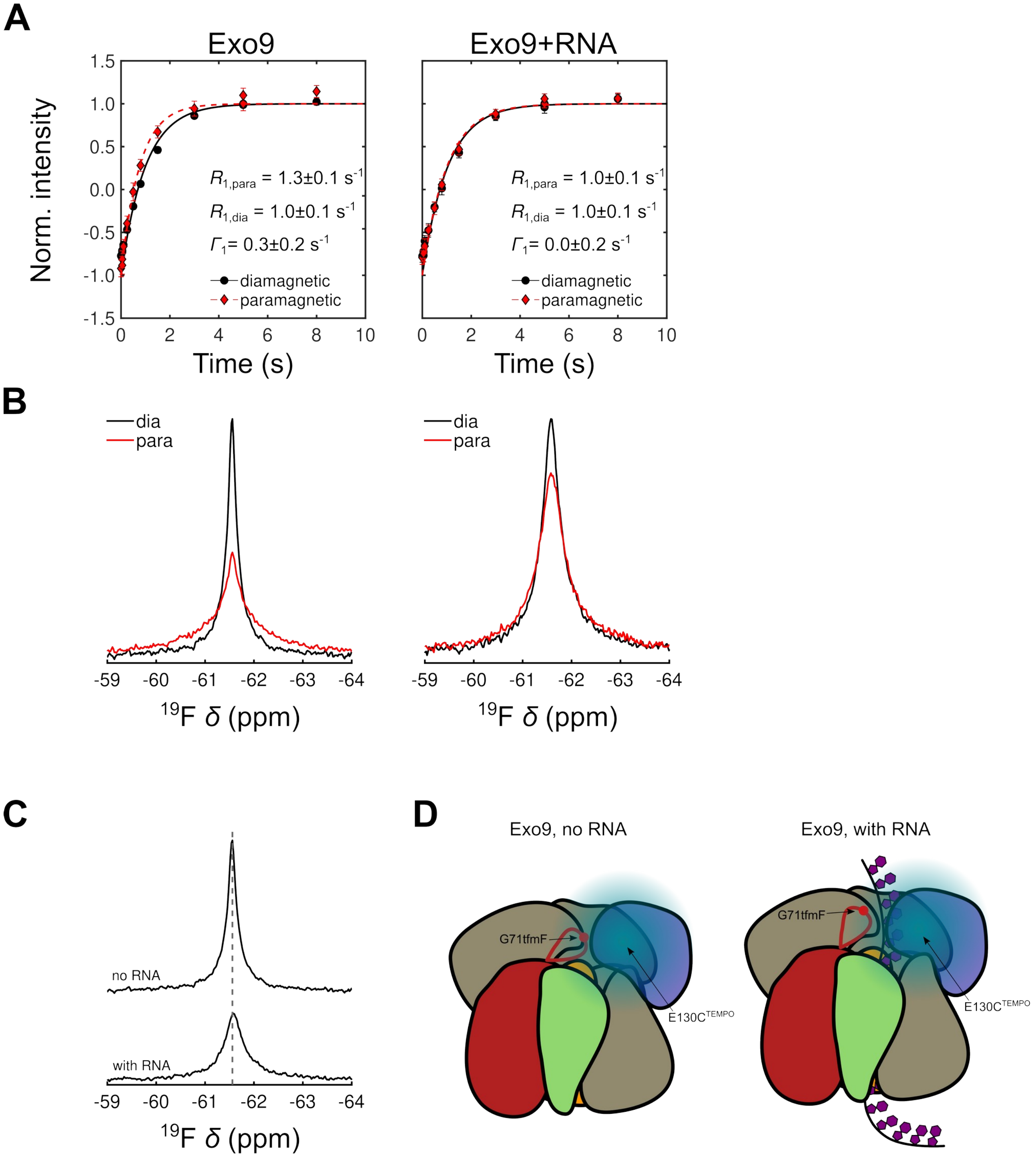

Figure S8: Location and RNA interactions of the entry loop of Rrp41. (A) T_1 inversion recovery experiments measured for Rrp41^{G71tfmF} Csl4$^{C122S,\ E130C\text{-}TEMPO}$ in Exo9 without (left) and with (right) RNA for paramagnetic (red) and diamagnetic (black) samples. R_1 relaxation rates and Γ_1 PREs derived from Eq. 1 and Eq. 3 are indicated in the panels (see table S3A). The spin-label gives rise to enhanced R_1 rates in the absence of RNA. For Γ_1 the effect is not statistically significant due to error propagation. Error bars are ±1 SD and were estimated from the signal-to-noise ratio of the NMR spectrum. Errors for R_1 were estimated from the fit using Matlab's nlparci function and errors for Γ_1

were estimated via error propagation. Errors are ±1 SD. (**B**) ^{19}F 1D experiments measured for Rrp41^{G71tfmF} Csl4$^{C122S,\ E130C\text{-}TEMPO}$ in Exo9 without (left) and with (right) RNA for paramagnetic (red) and diamagnetic (black) samples. (**C**) Overlay of the diamagnetic 1D ^{19}F spectra from panel **B** without (top) and with (bottom) RNA. The dotted line indicates the center of the resonance in the absence of RNA. (**D**) Schematic of the position of the entry loop of Rrp41 (red, the position of G71tfmF is shown as a red dot) with respect to Csl4 (blue, the PRE effect of the TEMPO spin-label at E130C is shown as a blue sphere). See also fig. S1C. In the absence of RNA the loop approaches Csl4 (left); in the presence of RNA the loop is displaced away from Csl4 (right). Rrp42 is shown in green and Rrp45 is depicted in orange.

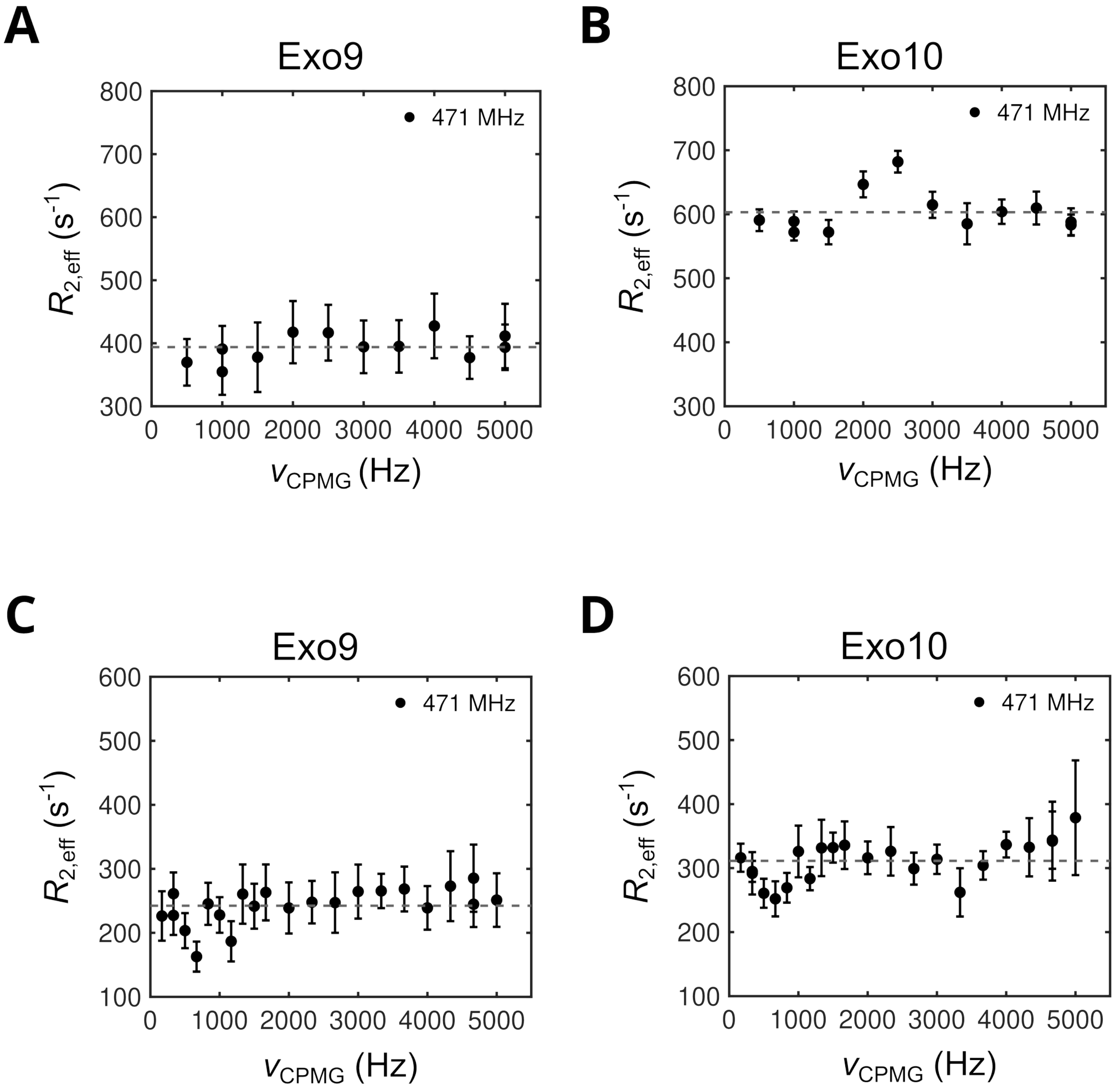

Figure S9: Relaxation dispersion experiments of the exit and entry loop. ^{19}F CPMG relaxation dispersion profiles for Rrp41^{D113tfmF} in (**A**) Exo9 and (**B**) Exo10 and CPMG relaxation dispersion profile for Rrp41^{Q86tfmF} in (**C**) Exo9 and (**D**) Exo10. Error bars are ±1 SD and estimated from the signal-to-noise ratio of the NMR spectrum.

A

ctRrp41	51	CVVTGPSEP*GP**RR**GTGAGTT**G**GGGAGGAGGGGSGG**Q***GKEAEVV	93
ssRrp41	56	AAVYGPKEMHP**RH**LSLP--------------------DRAVLR	75
scRrp41	51	TLVKGPKEPRL**KS**QMDT--------------------SKALLN	73
hsEXOSC4	50	AVVYGPHEIRG**SR**ARAL-------------------PDRALVN	73

B

ctRrp41	94	VSIV**I**AGFSSVDR*KR-HGR*N**DKRII**	117
ssRrp41	76	VRYHMTPFSTDERKN--PAPS**RR**EI	101
scRrp41	74	VSVNITKFSKFERSKSSHKNE**RR**VL	98
hsEXOSC4	74	CQYSSATFSTGERKRRP-HGD**RK**SC	97

C

ctRrp45	99	TEVLLS**R**LLE**K**T**I**R	112
ssRrp42	106	NAIELA**R**VVD**R**SLR	119
scRrp45	100	DEVLCS**R**IIE**K**SVR	113
hsEXOSC9	98	LLVKLN**R**LME**R**CLR	111

Figure S10: Sequence alignments of ctRrp41 and ctRrp45. Sequence alignment of (**A**) the entry loop of ctRrp41, (**B**) the exit loop of ctRrp41 and (**C**) a pore-facing helix of ctRrp45 with homologs from the archaeon *Sulfolobus solfataricus* (ssRrp41/ssRrp42), *S. cerevisiae* (scRrp41/scRrp45) and human (hsEXOSC4/hsEXOSC9). Amino acids that have previously been shown to coordinate RNA in *S. solfataricus* or *S. cerevisiae* (3, 4) and corresponding amino acids in *C. thermophilum* and human are in bold. Italicized residues are not visible in the cryo-EM structure. Positions G71, Q86 and D113, at which a tfmF-label was introduced, are highlighted in red, and Ile residues that show CSPs upon RNA addition to the exosome are shown in blue.

```
ctRrp42        70 VKAEVEKTVSRSKEDEEVGLLVASAGDMDVDDEEGYAKVGADNRTGEASWVEITVE 125
hsEXOSC7       62 VKAEMGTPKLEKPNEGYLEFFVDCS-------------------------ASATPE  92
scRrp42        62 IKSKVVDHHV---E---------------------------------NELLQVDVD  81
conservation      :*:::        :                                     .   :
```

Figure S11: Conservation of Rrp42-EL. Sequence alignment of an extended loop in ctRrp42 (Rrp42-EL) with the human (hsEXOSC7) and *S. cerevisiae* (scRrp42) homologs.

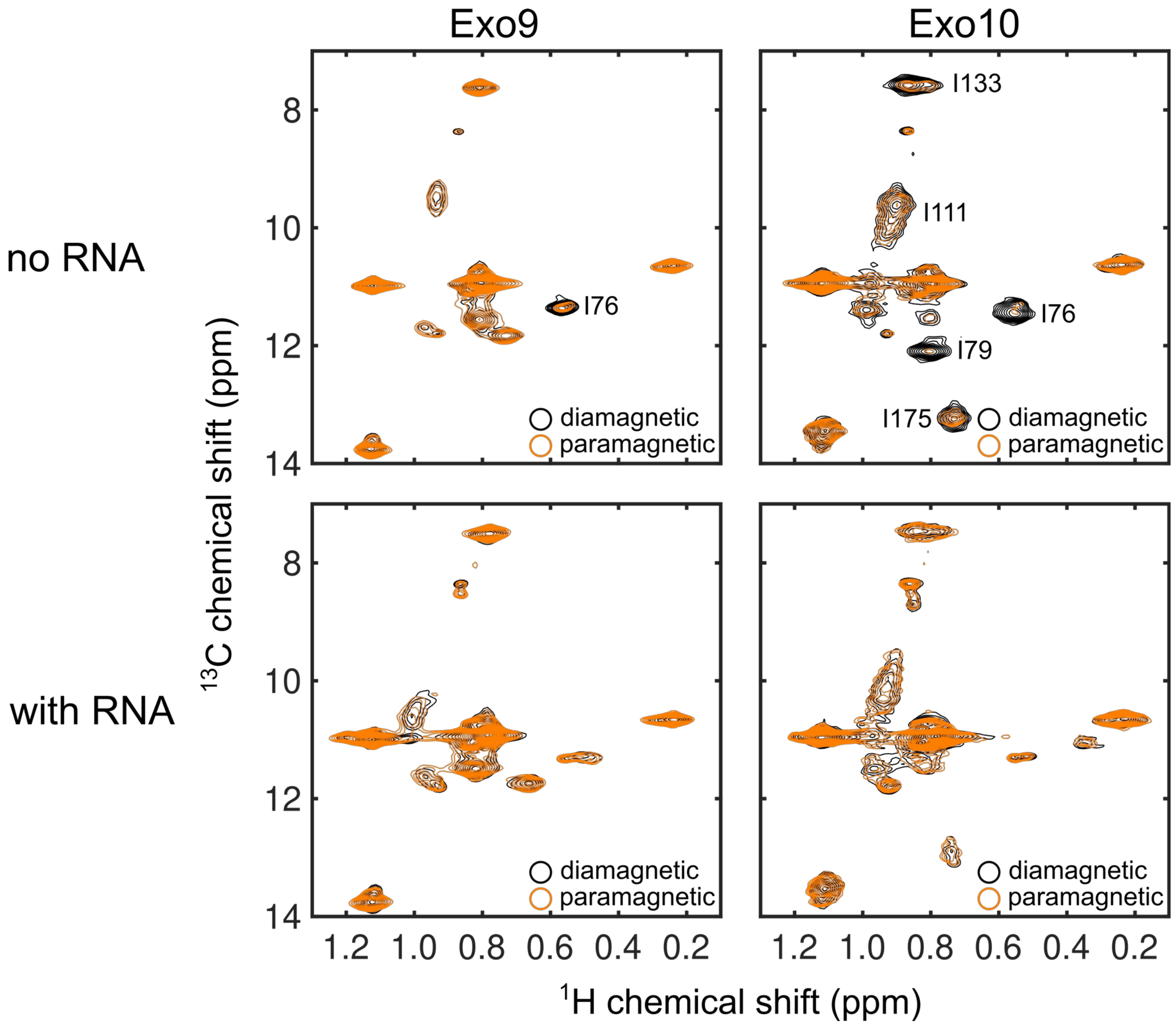

Figure S12: Methyl PREs of Rrp42$^{C59S, A106C\text{-}TEMPO}$. PREs for Ile-δ1 of Rrp45 in Exo9 and Exo10 with and without RNA. The spectrum of the diamagnetic sample is shown in black and the spectrum of the paramagnetic sample is depicted in orange. Resonances of residues that exhibit a substantial PRE ($I_{para}/I_{dia} < 0.7$) are labeled.

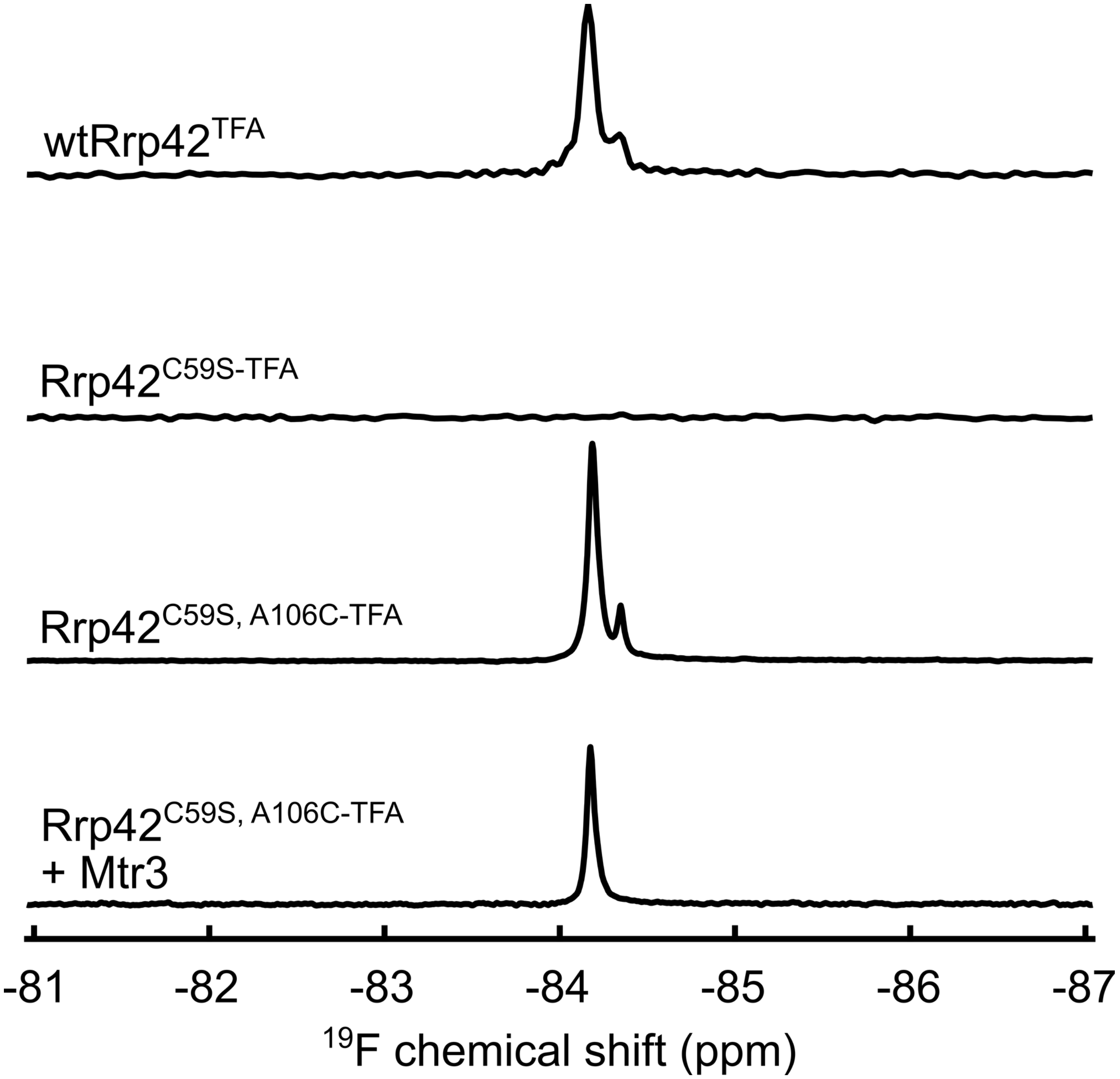

Figure S13: TFA-labeling of Rrp42 mutants. ^{19}F spectra of TFA-labeled wtRrp42 (row 1), Rrp42^{C59S} (row 2), Rrp42$^{C59S, A106C}$ (row 3) and Rrp42$^{C59S, A106C}$ in complex with Mtr3 (row 4).

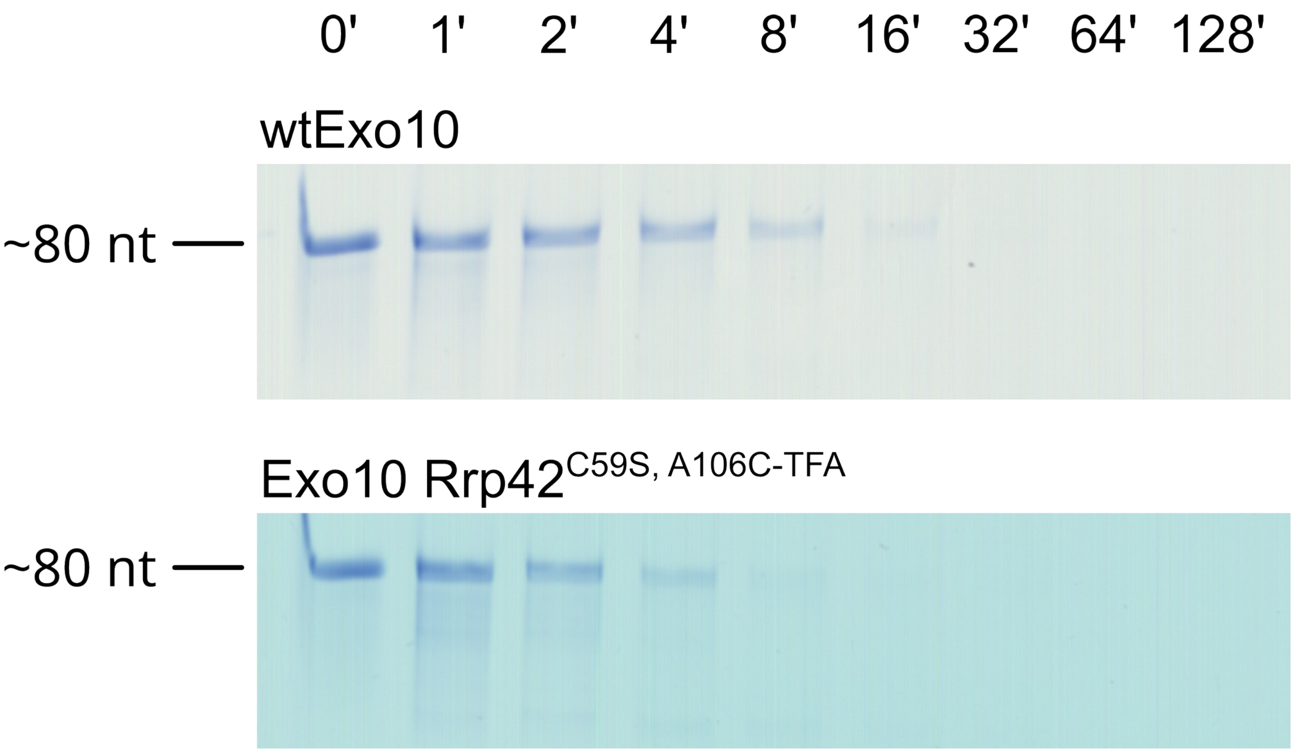

Figure S14: Activity assay of Exo10 variants. Activity assay of wtExo10 (top) and Exo10 Rrp42$^{C59S, A106C-TFA}$ (bottom) using an 80mer RNA (see table S7). The RNA substrate is degraded with similar rates in wtExo10 complex and the Exo10 complex that is ^{19}F labeled in Rrp42-EL. The difference in degradation rates observed is within the measurement uncertainty of the qualitative evaluation.

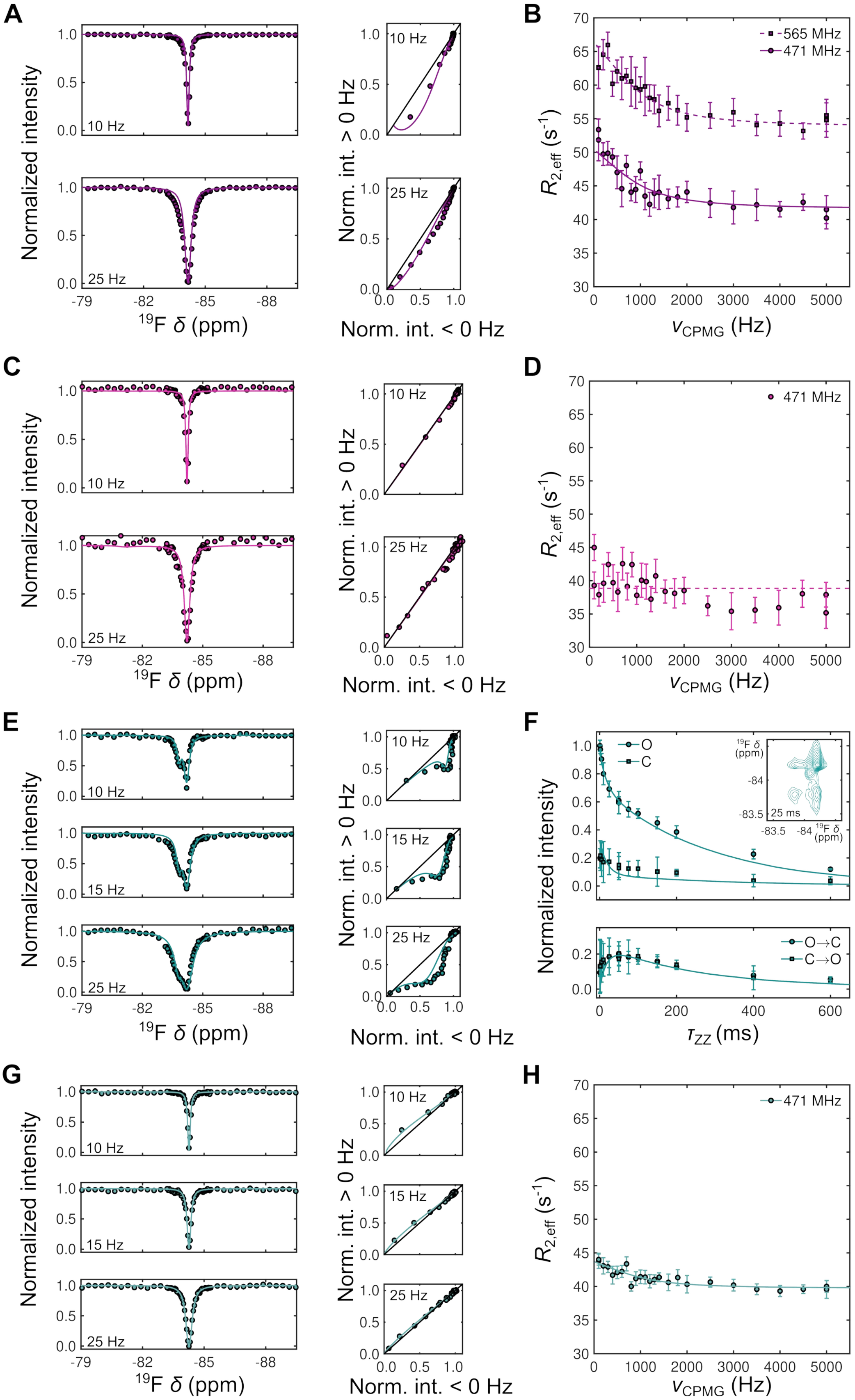

A
B
C
D
E
F
G
H
Normalized intensity
10 Hz
15 Hz
25 Hz
^{19}F δ (ppm)
-79
-82
-85
-88
Norm. int. > 0 Hz
Norm. int. < 0 Hz
$R_{2,\mathrm{eff}}$ (s^{-1})
ν_{CPMG} (Hz)
565 MHz
471 MHz
O
C
O→C
C→O
25 ms
τ_{ZZ} (ms)

Figure S15: Exchange dynamics of Rrp42-EL. (**A**) and (**B**): Dynamics of Rrp42$^{C59S, A106C\text{-}TFA}$ in Exo9. (**A**) CEST experiments (left panels) and symmetry of the profile's dip (right panels). (**B**) CPMG RD profiles. (**C**) and (**D**): Dynamics of Rrp42$^{C59S, A106C\text{-}TFA}$ in Exo9 with RNA. (**C**) CEST experiments (left panels) and symmetry of the profile's dip (right panels). (**D**) CPMG RD profile. (**E**) and (**F**): Dynamics of Rrp42$^{C59S, A106C\text{-}TFA}$ in Exo10. (**E**) CEST experiments (left panels) and symmetry of the profile's major dip (right panels). (**F**) EXSY profiles for diagonal (open state 'O', closed state 'C') and cross (O→C, C→O) peaks. The inset shows the $^{19}F/^{19}F$ correlation spectrum for τ_{ZZ} = 25 ms. (**G**) and (**H**): Dynamics of Rrp42$^{C59S, A106C\text{-}TFA}$ in Exo10 with RNA. (**G**) CEST experiments (left panels) and symmetry of the profile's dip (right panels). (**H**) CPMG RD profile. A two-site exchange model as described in the methods section was globally fitted to the data in panels **A**, **B**, **E** and **F**. Fitted parameter values are shown in table S4.

The symmetry of the main CEST dip is assessed by plotting intensities that are equidistant from the main dip (at 0 Hz) against each other, where offsets < 0 Hz are on the x-axis and the respective offsets > 0 Hz are on the y-axis. If all data points are located on a 45° line, the main dip is symmetric and the CEST experiment does not report on dynamics (as in **C**). If data points are not located on a 45° line, the main dip is asymmetric due to CEST-observable exchange dynamics (as in **A** and **E**). This approach allows to determine exchange dynamics even in the absence of a pronounced exchange dip (as in **A**) (5).

Error bars in **B**, **D**, **F** and **H** are ±1 SD and estimated from the signal-to-noise ratio of the NMR spectrum.

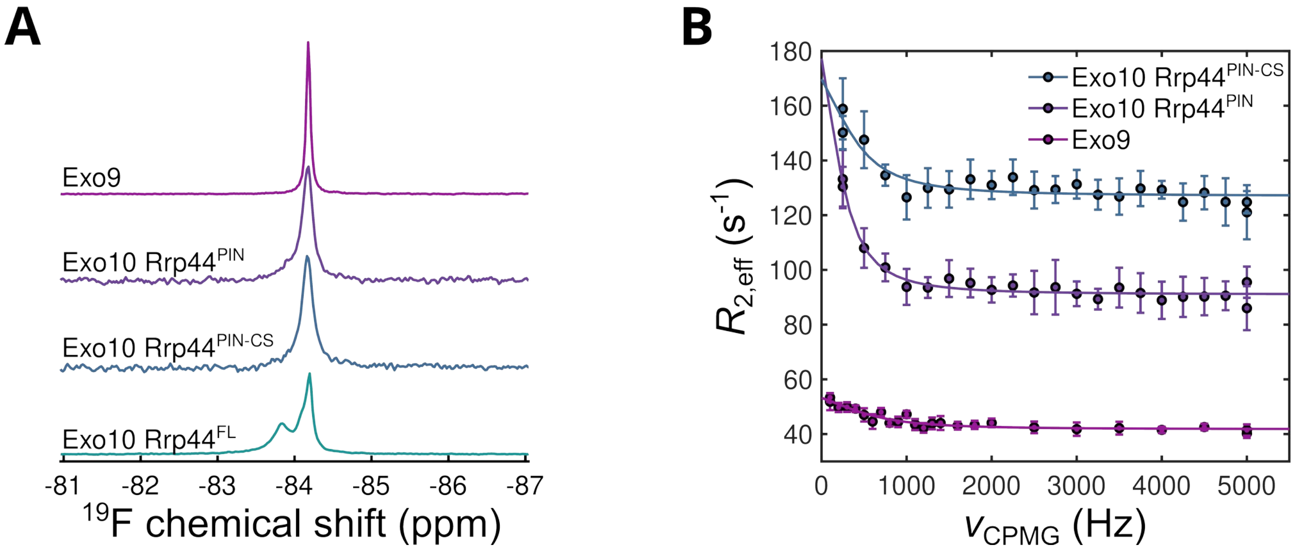

Figure S16: Interaction of Rrp42-EL with Rrp44. (**A**) ^{19}F spectra of Rrp42$^{C59S, A106C\text{-}TFA}$ in Exo9, Exo10 Rrp44^{PIN}, Exo10 Rrp44^{PIN+CS} and Exo10 with full-length Rrp44. (**B**) CPMG relaxation dispersion profiles for Exo10 Rrp44^{PIN+CS}, Exo10 Rrp44^{PIN} and Exo9. Note that CPMG relaxation dispersion experiments on the Exo10 complex (with full-length Rrp44) suffered from very fast relaxation rates that prevented us from obtaining high quality fluorine NMR data. Error bars are ±1 SD and estimated from the signal-to-noise ratio of the NMR spectrum.

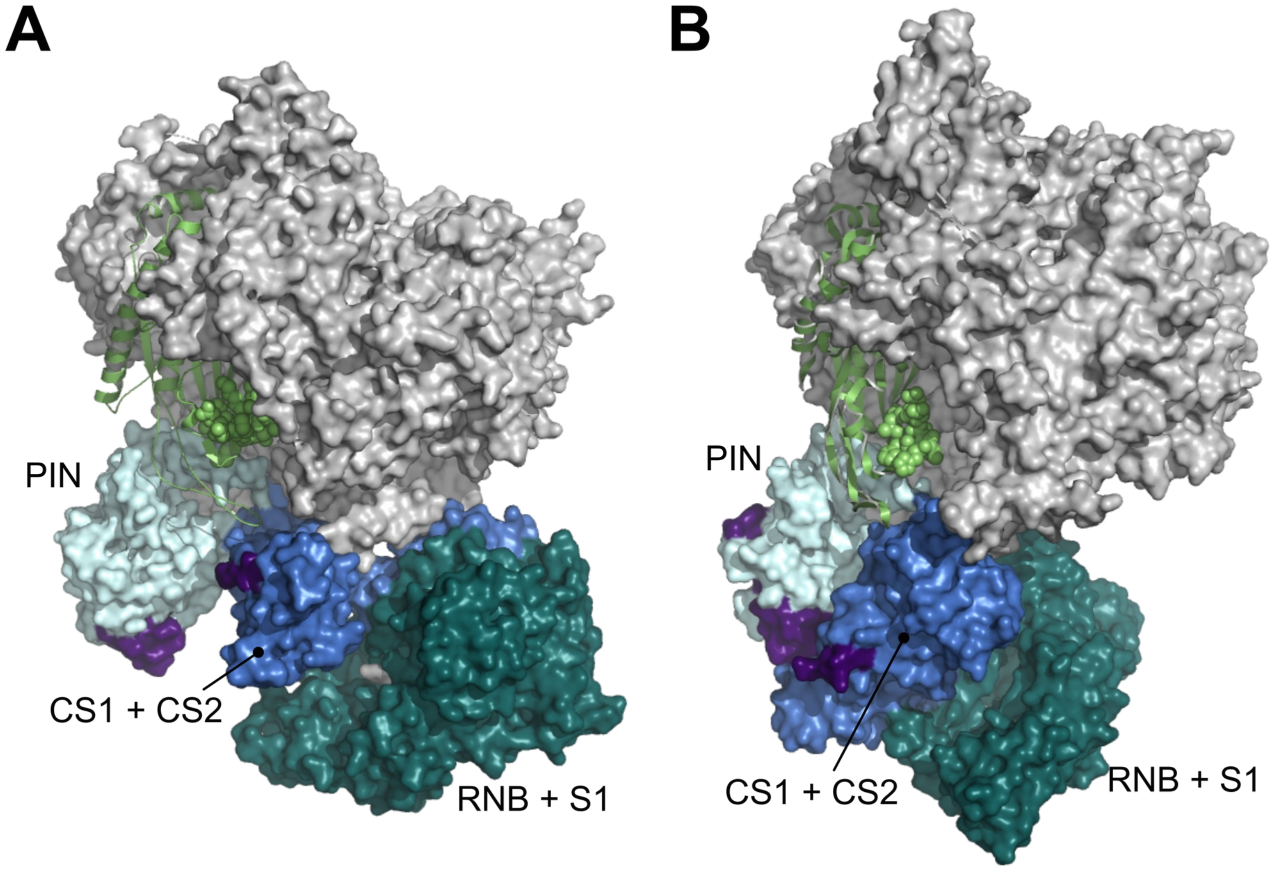

Figure S17: Localization of Rrp42-EL with respect to Rrp44 in yeast and human exosome complexes. Structures of (**A**) the human (PDB ID: 6H25 (2)) and (**B**) the *S. cerevisiae* (PDB ID: 4IFD (6)) exosome. Exo9 is shown in gray surface representation, except for Rrp42, which is displayed in green cartoon. Residues in the loop corresponding to ctRrp42-EL (see fig. S11) are green and shown in spherical representation. Rrp44 domains are displayed in colored surface representation: the PIN domain is in light blue, the CS1 and CS2 domains are in marine and the RNB and S1 domains are in dark teal. The structurally resolved sections of the connecting loop between the PIN and CS1 domain is highlighted in purple. The CS1 and CS2 domains come close to Rrp42, and are kept in place there due to interactions between the bottom of the Exo9 barrel and the Rrp44 RNB-S1 domains. See also fig. S24.

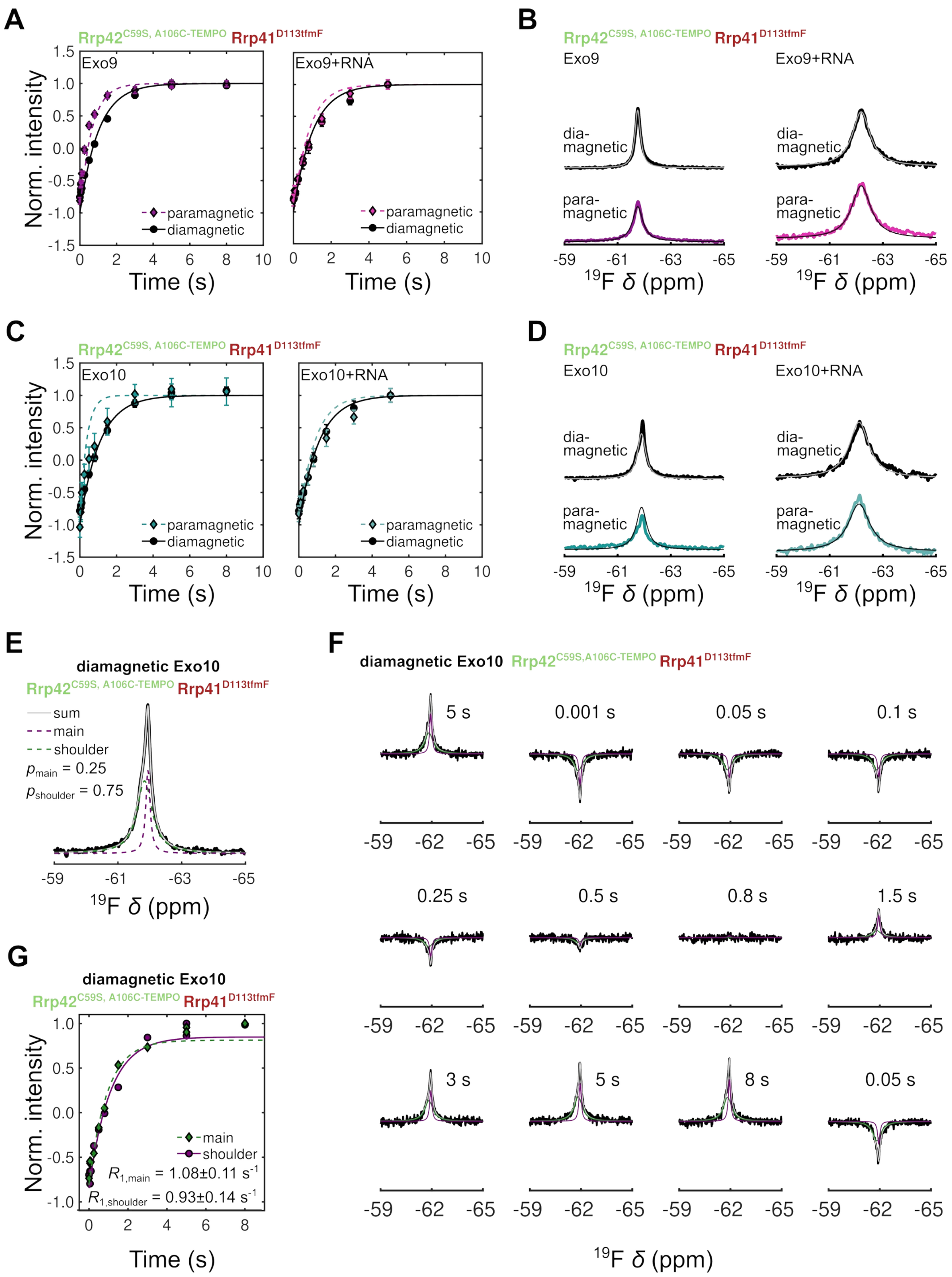

Figure S18: Fast loop dynamics of Rrp42-EL. (**A**) T_1 inversion recovery experiments and (**B**) ^{19}F 1D spectra for Rrp41$^{\mathrm{D113tfmF}}$ in Exo9 Rrp42$^{\mathrm{C59S,\ A106C\text{-}TEMPO}}$ without (left panel) and with (right panel) RNA for paramagnetic (in color) and diamagnetic (black) samples. (**C**) T_1 inversion recovery experiments and (**D**) ^{19}F 1D spectra for Rrp41$^{\mathrm{D113tfmF}}$ in Exo10 Rrp42$^{\mathrm{C59S,\ A106C\text{-}TEMPO}}$ without (left panel) and with (right panel) RNA for paramagnetic (in color) and diamagnetic (black) samples. A two-site exchange model as described in the methods section was globally fitted to the data. Fitted parameter values are shown in table S4. (**E**) Double Lorentzian fit (green, purple) to the 1D ^{19}F

spectrum of diamagnetic Exo10 Rrp42$^{C59S, A106C\text{-}TEMPO}$ Rrp41^{D113tfmF}. The sum of the two component fit is in grey. Populations of the conformations are indicated in the figure. (**F**) Double Lorentzian fit (green, purple) to T_1 data of diamagnetic Exo10 Rrp42$^{C59S, A106C\text{-}TEMPO}$ Rrp41^{D113tfmF}. The sum of the two component fit is in grey. Inversion recovery delays are indicated for each spectrum. (**G**) Exponential fits (Eq. 1) to intensities obtained from **F**. R_1 rates obtained from the fits are indicated in the figure. Error bars in **A** and **C** are ±1 SD and estimated from the signal-to-noise ratio of the NMR spectrum. Error bars for relaxation rates in **G** are ±1 SD and were estimated from the fit using Matlab's nlparci function.

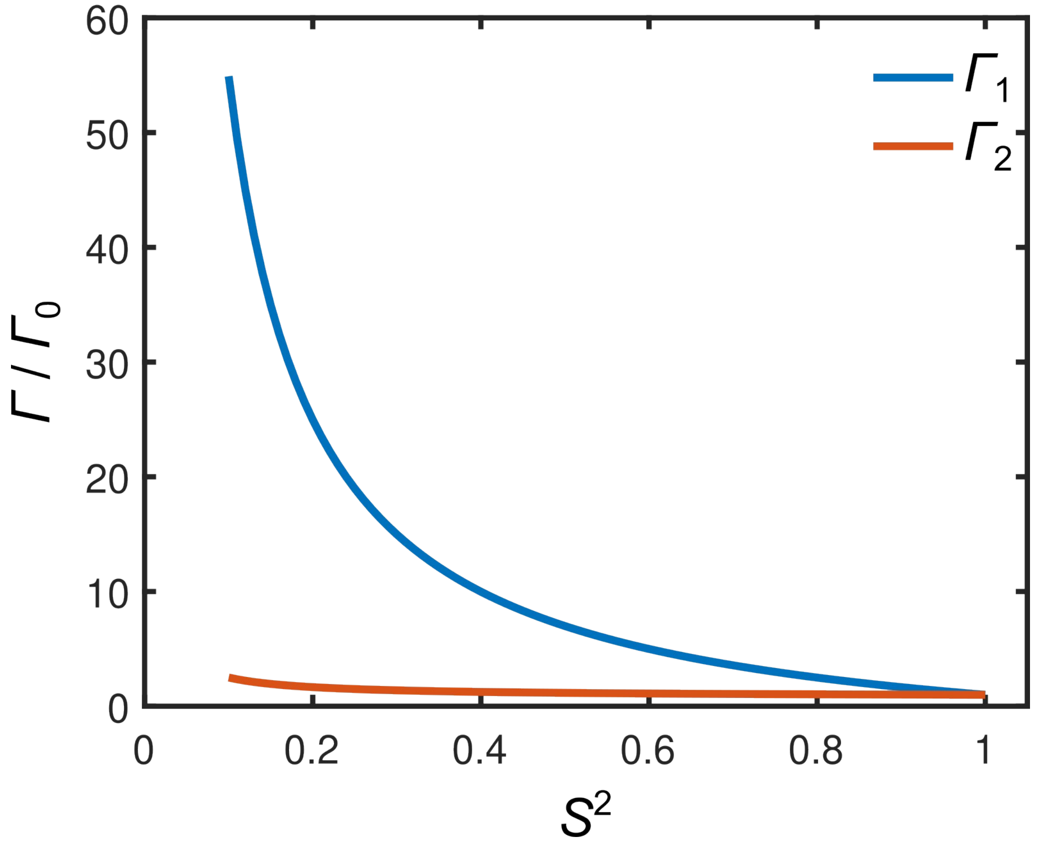

Figure S19: Dependency of the PRE rates Γ_1 and Γ_2 on the order parameter S^2. $\Gamma_{(1\text{: blue or }2\text{: red})}$ are calculated based on Eq. 9 (using τ_i = 10 ns, τ_r = 100 ns and $\omega = 2*\pi*470*10^6$ rad/s; corresponding to the fluorine resonance frequency on a 500 MHz NMR spectrometer). Γ_0 is the relaxation rate for S^2 = 1. The plot indicates that Γ_1 depends strongly on S^2, whereas Γ_2 is largely unaffected by the order parameter.

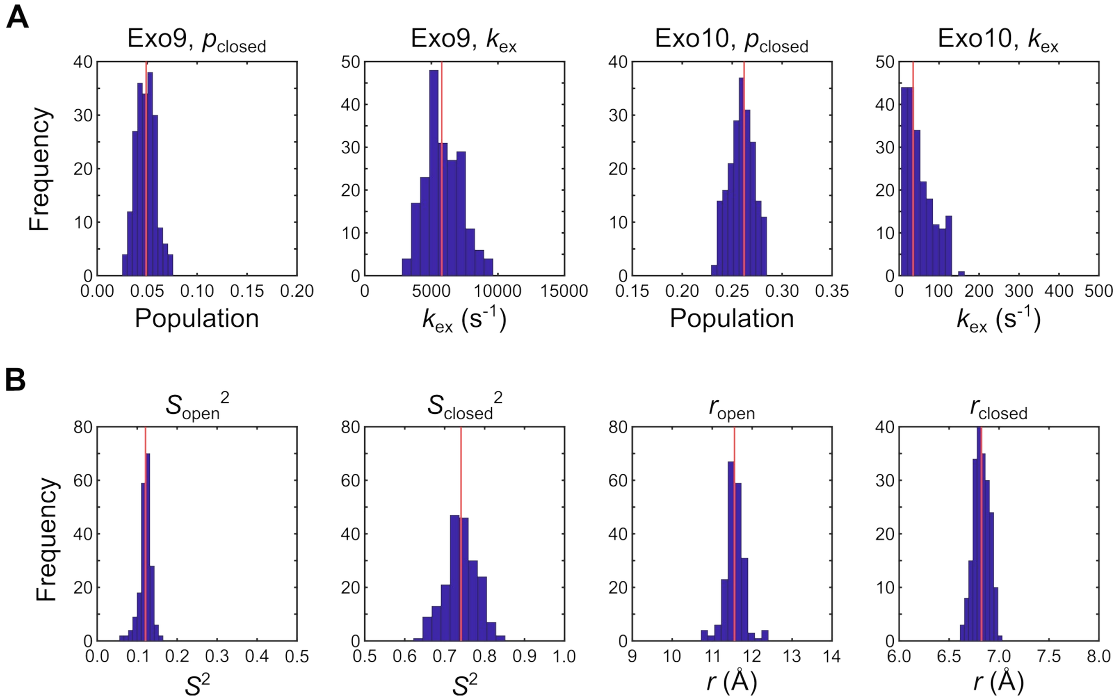

Figure S20: Monte-Carlo simulations of dynamics data. 200 datasets were simulated and a two-site exchange model was fitted to these artificial datasets to extract parameter uncertainties as described in the method section. The histograms show the distribution of fitted values (**A**) for the population of the closed state (p_{closed}) and exchange rate (k_{ex}) in Exo9 and Exo10 and (**B**) for the order parameter (S^2) and distance (r) between the ^{19}F label (Rrp41^{D113tfmF}) and the paramagnetic center of Rrp42$^{C59S, A106C-TEMPO}$ of the open and closed state of Rrp42-EL. The red line indicates the parameter value obtained from the best fit of the two-site exchange model to the experimental data.

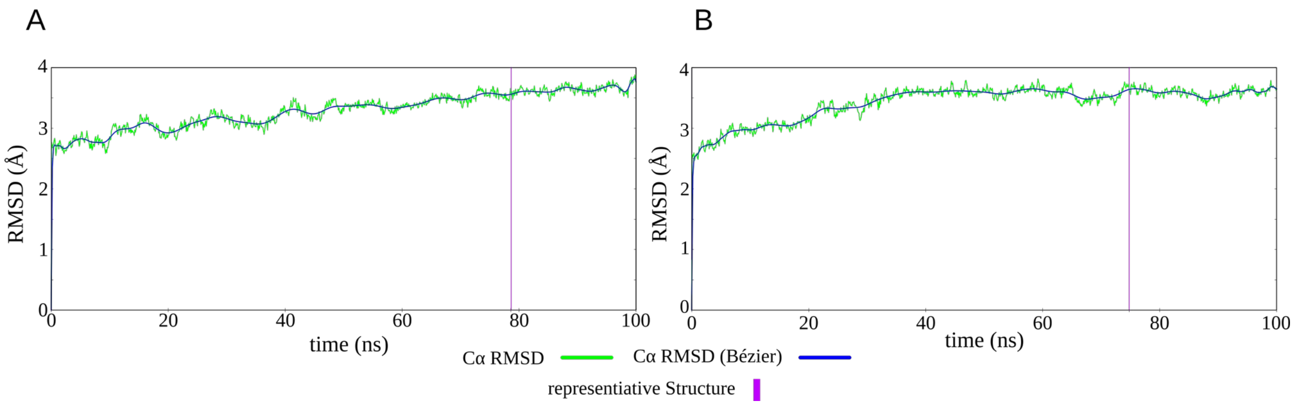

Figure S21: C$^{\alpha}$ root mean square deviation (RMSD). The plot of the RMSD of the C$^{\alpha}$ carbon atoms of the complete simulation system over the course of the MD simulation of the Rrp42-EL closed (left) and open (right) state with respect to the MD starting structure converges towards a plateau. Therefore, both RMSD plots indicate that an energetically equilibrated structure is reached. Highlighted by the magenta line is the simulation frame that constitutes the representative structure of the equilibrated part of each simulation.

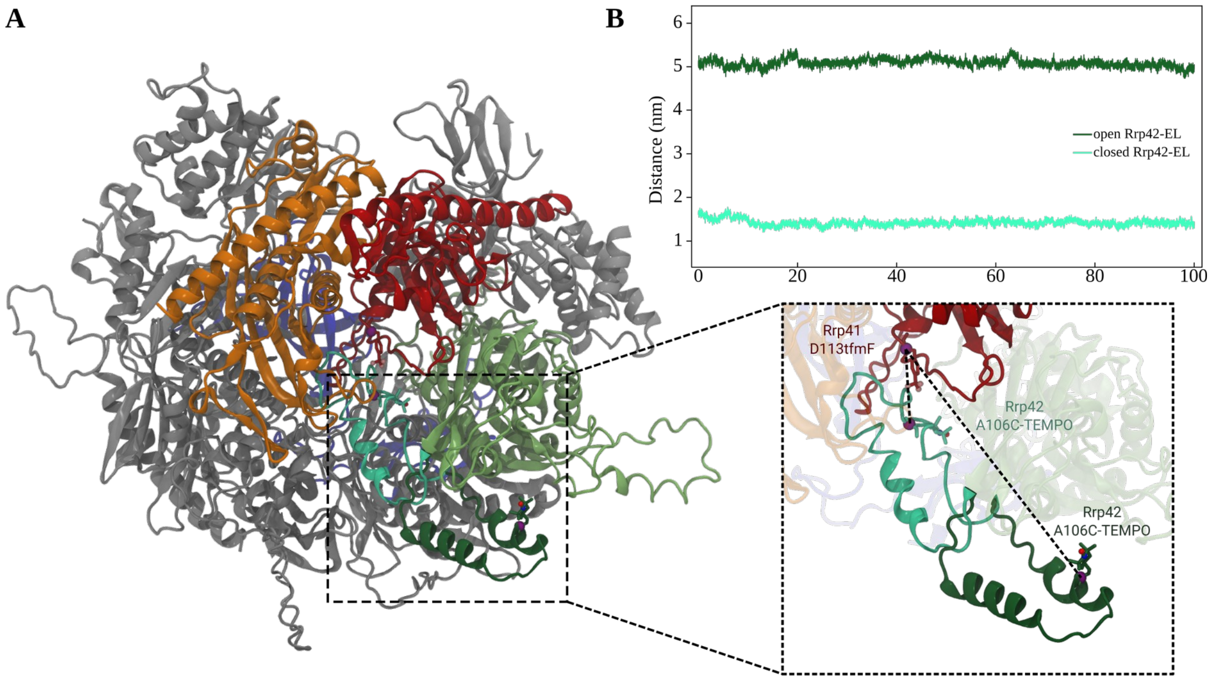

Figure S22: Distances between the residues labeled for ^{19}F PRE experiments. (**A**) Location of the ^{19}F and spin label within the modeled starting structures of Exo9 with open state (dark green) and closed state (cyan) Rrp42-EL. Rrp42 is shown in green, Rrp41 in red, Rrp45 in orange and Csl4 in blue. Zoom: Distance of the C^{α} carbon atoms of the labeled residues within the starting model of the MD simulation. (**B**) Measured distance between the C^{α} atoms of the residues labeled in the ^{19}F PRE experiments (Rrp42-A106 and Rrp41-D113) within the MD simulation trajectories of the open (cyan) and closed state (dark green). Within the MD simulations neither the spin label nor the D113tfmF mutation are incorporated. The open and closed state show a clear difference in the C^{α} distances which is qualitatively comparable to the experimentally observed differences in the spin label to tfmF distance (Fig. 4, Fig S18).

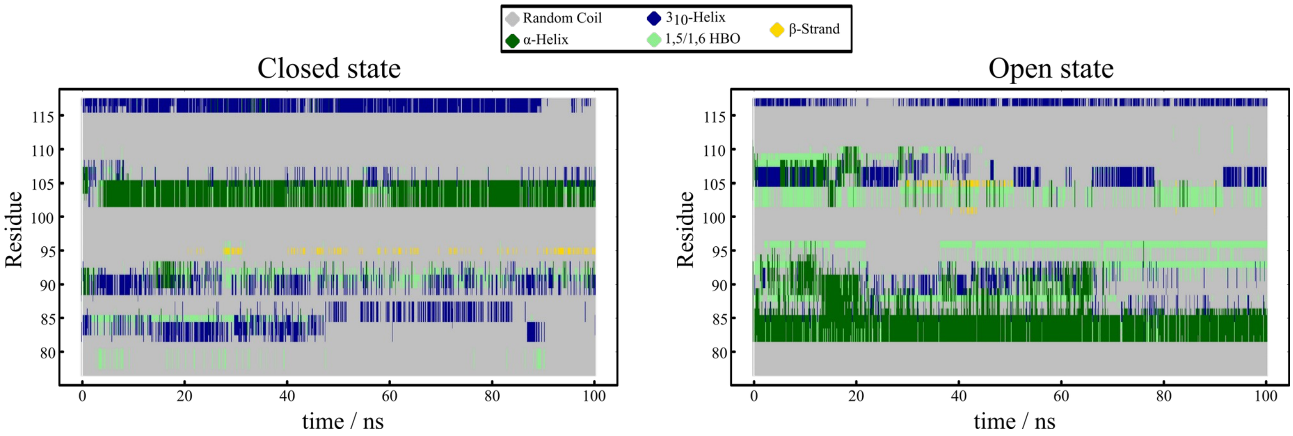

Figure S23: Comparison of the secondary structure of Rrp42-EL within the MD simulations of the closed and open state. Each residue of Rrp42-EL is colored with respect to its secondary structure over the time course of the MD simulation with a step size of 0.1 ns. The plot differentiates between random coil (grey), α-helix (dark green), 3_{10}-helix (blue), β-strand (yellow) and 1,5 or 1,6 hydrogen bonds (light green). The separated first column shows the secondary structure of the starting structure obtained from the AlphaFold prediction. The MD simulations indicate that parts of the predicted α-helices are not stable within the simulations. They might fluctuate between α-helix and random coil or there might be less α-helical content than predicted.

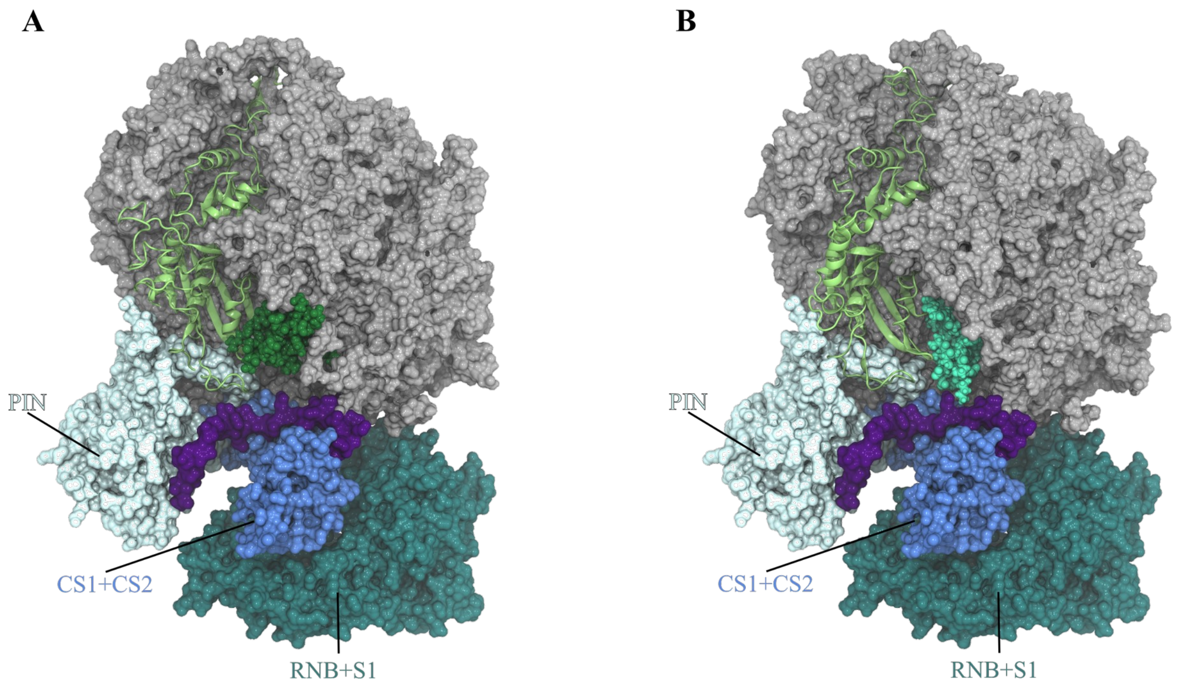

Figure S24: Localization of Rrp42-EL with respect to Rrp44. Superposition of the representative open (**A**) and closed (**B**) state of Rrp42-EL from ctExo9 complemented with Rrp44 from *S. cerevisiae* (chain J, PDB: 4IFD (6)). Exo9 is shown in surface representation in grey, Rrp42 as cartoon green, open Rrp42-EL in dark-green and closed Rrp42-EL in light green, each displayed as spheres. Rrp44 is shown in surface representation, the PIN domain is colored light blue, CS1 and CS2 are colored marine, the RNB and S1 domains are shown in dark teal. The connective loop between the PIN and CS1 domain is highlighted in purple. See also fig. S17.

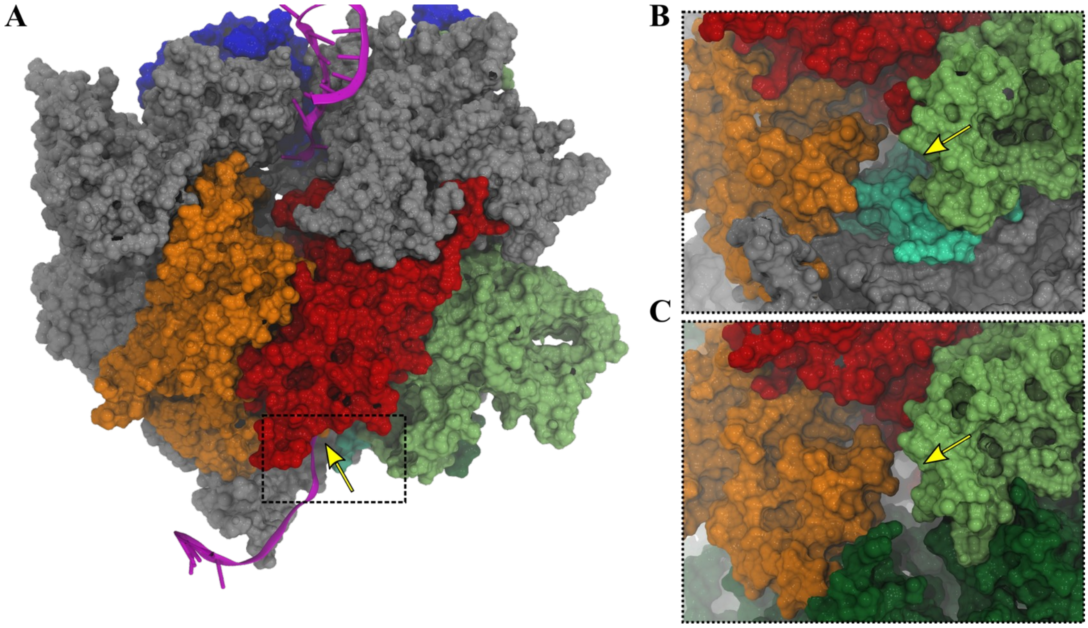

Figure S25: The closed state of Rrp42-EL blocks the RNA channel. (**A**) The RNA (purple) is modeled into the representative structures of the open and closed state of the Exo9 MD simulations. RNA positioning was guided by the X-Ray structure of *S. cerevisiae* Exo9 (PDB ID: 4IFD (6)). (**B**) The enlargement of the exit tunnel with removed RNA illustrates the blocking of Rrp42-EL in the closed state (cyan) as indicated by the arrow. (**C**) In the open state the exit tunnel is free as Rrp42-EL (dark green) is remote from the exit site of the RNA channel. Subunit coloring: Csl4: blue, Rrp41: red, Rrp45: orange, Rrp42: green.

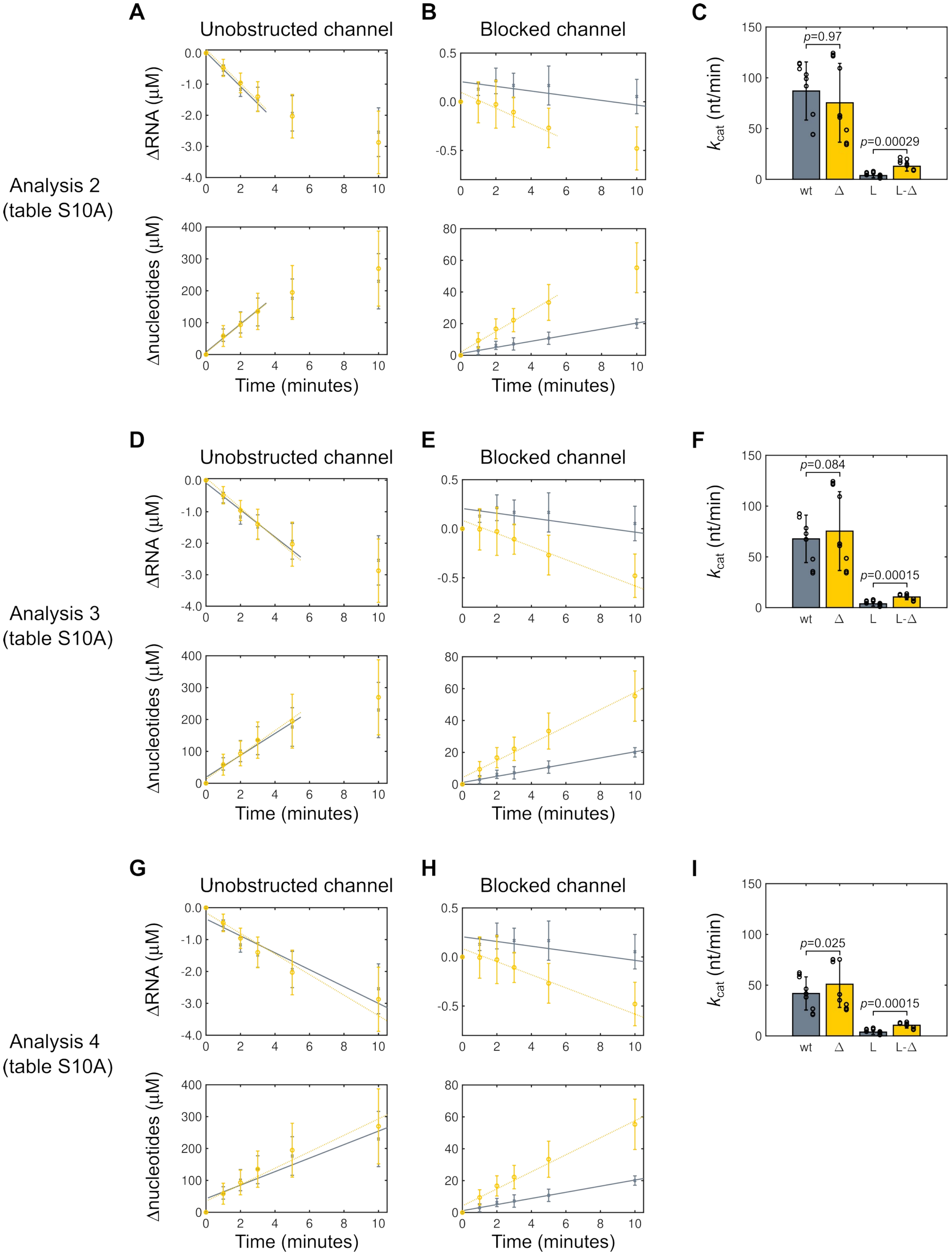

Figure S26: Extended analysis of exosome activity data. HPLC-based activity assays using an 80mer RNA (see table S7) (**A, D, G**) for wtExo10 (gray crosses, solid line) and Exo10 Rrp42$^{\Delta 93\text{-}125}$

(yellow circles, dashed line) and (**B, E, H**) for channel-blocked Exo10 Rrp45-L (gray crosses, solid line) and Exo10 Rrp45-L Rrp42$^{\Delta 93\text{-}125}$ (yellow circles, dashed line). (**C, F, I**) Catalytic activity of exosome constructs. wt = wtExo10, Δ = Exo10 Rrp42$^{\Delta 93\text{-}125}$, L = Exo10 Rrp45-L, L-Δ = Exo10 Rrp45-L Rrp42$^{\Delta 93\text{-}125}$. *p*-values are derived from a two-sided, paired-sample *t*-test. (**A, B, C**) Analysis 2 in table S10B. (**D, E, F**) Analysis 3 in table S10B. (**G, H, I**) Analysis 4 in table S10B. The lines are global linear fits to the linear activity regime as specified in table S10B. Data were obtained from three biological replicates with three technical repeats. Error bars are ±1 SD.

A

```
ctRrp45       79 IATELSPMTSPTFEVNRPT-ETEV 101
scRrp45       79 ISTEISPMAGSQFENGNITGEDEV 102
hsEXOSC9      78 FNLELSQMAAPAFEPGRQS-DLLV 100
conservation     :  *:* *:.  ** .. : :  *
```

B

```
ctRrp45       79 IATELSPMTSPTFEVNRPTETEV 101
cryo-EM          SSSSS-------------HHHHH
```

C

```
scRrp45       79 ISTEISPMAGSQFENGNITGEDEV 102
PDB 4IFD:A       SS----HHH-----------HHHH
```

Figure S27: Characteristics of a channel facing loop in Rrp45. (**A**) Sequence alignment of a channel facing loop in Rrp45 with the *S. cerevisiae* (scRrp45) and human (hsEXOSC9) homologs. The loop extension was introduced between residues N94 and R95 highlighted in bold. (**B**) Secondary structure of the loop from ctRrp45 based on the cryo-EM structure. (**C**) Secondary structure of the loop from scRrp45 obtained from PDB ID: 4IFD, chain A. S = strand, H = helix, - = unstructured.

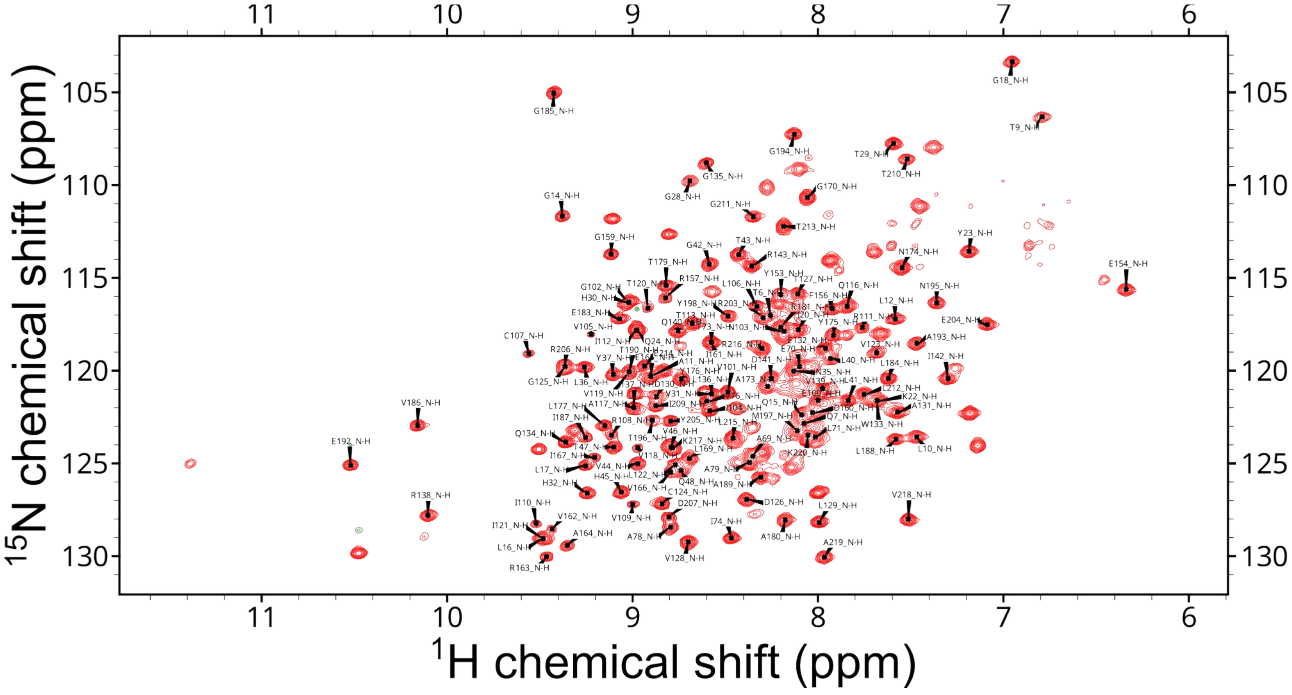

Figure S28: ^{15}N-^{1}H TROSY of Csl4. Assigned resonances are labeled.

Table S1: X-ray crystallography and cryo-EM data collection and refinement statistics. Statistics for the *C. thermophilum* core exosome (ctExo9) structure obtained by (**A**) X-ray crystallography (PDB ID: 8PEL) and (**B**) single particle cryo-EM (PDB ID: 8R1O, EMDB ID: EMD-18825). Statistics for the highest-resolution shell in the X-ray structure are shown in parentheses.

A

**Parameter**	**Value**
**Data collection**	
Space group	P $2_1 2_1 2_1$
Cell dimensions	
a, b, c (Å)	100.525, 148.383, 195.065
α = β = γ (°)	90, 90, 90
Wavelength (Å)	0.9999
Resolution range (Å)	48.77 – 3.81 (3.946 – 3.81)
Total reflections	393908 (37705)
Unique reflections	29143 (2793)
Completeness (%)	99.57 (97.14)
Redundancy	13.5 (13.5)
$I/\sigma(I)$	5.01 (1.19)
$CC_{1/2}$	0.984 (0.586)
CC^{*}	0.996 (0.86)
R_{merge}	0.5272 (2.127)
R_{meas}	0.5479 (2.211)
R_{pim}	0.1481 (0.5971)
**Refinement**	
Resolution range (Å)	48.77 – 3.81
No. reflections	29114 (2790)
R_{work}	0.2427 (0.3486)
R_{free}	0.2923 (0.3830)
No. protein atoms (without H)	17656
Average B factor (Å^2)	103.52

R.m.s. deviations	
Bond lengths (Å)	0.004
Bond angles (°)	0.690
Ramachandran plot	
Favored (%)	97.64
Allowed (%)	2.32
Outliers (%)	0.04
Rotamer outliers (%)	0.05
Clashscore	14
MolProbity score	1.29

B

**Parameter**	**Value**
**Data collection and processing**	
Microscope	JEOL CryoArm
Voltage (kV)	200
Camera	Gatan K2 Summit
Magnification	60000
Pixel size (Å)	0.772
Total electron exposure (e^-/Å^2)	40
No. frames collected during exposure	40
Defocus range (µm)	0.6 – 2.2
Energy filter slit width (eV)	20
No. micrographs collected	6579
No. micrographs used	6572
No. extracted particles	2448810
**Reconstruction**	
No. refined particles	1541277
No. particles used in the final reconstruction	276958
Point-group	C1
Resolution (FSC 0.143) (Å)	
Global (unmasked/masked)	3.19/2.99

Local	2.907 – 3.707
Map sharpening B factor (Å^2)	-114.7
**Refinement**	
Resolution (Å)	3.2
No. protein atoms (without H)	17918
No. protein residues	2335
Average B factor (Å^2)	125.57
R.m.s. deviations	
Bond lengths (Å)	0.006
Bond angles (°)	0.937
Ramachandran plot	
Favored (%)	97.60
Allowed (%)	2.40
Outliers (%)	0
Rotamer outliers (%)	0.56
Clashscore	4
MolProbity score	1.30
CC_{volume}	0.830

Table S2: Ile-δ1 and Met-ε1 assignments. Ile-δ1 and Met-ε1 resonance assignments of (**A**) Csl4, (**B**) Rrp41 and (**C**) Rrp45 as monomer and when reconstituted in Exo9 and Exo10. Note that multiple resonances for one residue are arbitrarily assigned as 'A' or 'B', i. e. A or B assignments for different resonances do not necessarily report on the same conformation. n.d.: not determined/ not assigned (e. g. due to severe signal overlap or resonance broadening beyond detection).

A

Resonance	monomer		in Exo9		in Exo10	
	^{1}H (ppm)	^{13}C (ppm)	^{1}H (ppm)	^{13}C (ppm)	^{1}H (ppm)	^{13}C (ppm)
Csl4-I18	0.981	11.734	1.023	12.117	1.022	12.111
Csl4-I60[1]	0.912	10.003	0.926	9.981	0.926	9.980
Csl4-I72	0.038	11.255	-0.002	10.656	-0.002	10.661
Csl4-I95[1]	0.912	10.003	0.926	9.981	0.926	9.980
Csl4-I102	0.828	6.792	0.810	7.330	0.812	7.326
Csl4-I108	0.822	9.135	0.845	9.297	0.842	9.289
Csl4-I110	1.012	11.000	1.171	11.837	1.162	11.830
Csl4-I119	0.707	11.182	0.628	10.991	0.631	11.026
Csl4-I135	0.236	10.833	0.275	11.010	0.273	10.984
Csl4-I140	0.921	12.351	0.958	12.327	0.956	12.317
Csl4-I159	0.765	6.465	0.903	6.590	0.898	6.634
Csl4-I165	0.876	11.237	0.888	11.238	0.883	11.214
Csl4-I185	1.003	12.883	1.078	12.729	1.078	12.730
Csl4-I207	0.828	7.858	0.880	7.850	0.881	7.847
Csl4-M1	2.161	14.535	2.203	14.454	2.206	14.452
Csl4-M195	1.492	15.104	n.d.	n.d.	n.d.	n.d.

[1] Csl4-I60 and Csl4-I95 overlap.

B

Resonance		monomer		in Exo9		in Exo10	
		^{1}H (ppm)	^{13}C (ppm)	^{1}H (ppm)	^{13}C (ppm)	^{1}H (ppm)	^{13}C (ppm)
Rrp41-I30	A	1.039	11.306	1.036	10.674	1.023	10.732
	B	1.022	11.116				
Rrp41-I96		0.872	12.765	n.d.	n.d.	n.d.	n.d.

Rrp41-I98		0.765	9.270	0.676	9.295	0.484	9.404
Rrp41-I116	A	0.855	8.743	0.858	8.650	0.695	7.570
	B	0.826	8.221				
Rrp41-I117[2]		0.947	9.522	0.961	9.749	0.939	9.712
Rrp41-I141	A	0.750	11.258	0.748	11.628	0.734	11.591
	B	0.716	11.333				
Rrp41-I143		0.560	11.137	0.554	11.288	0.549	11.286
Rrp41-I159	A	0.540	10.599	0.576	10.438	0.567	10.425
	B	0.540	10.495				
Rrp41-I171	A	0.778	10.465	0.659	10.877	0.637	10.687
	B	0.690	10.345				
Rrp41-I251[2]		0.969	9.890	0.977	9.608	0.939	8.987
Rrp41-I254		0.948	11.027	0.942	11.202	0.899	11.099
Rrp41-I267	A	0.897	9.825	0.793	11.005	0.782	11.073
	B	0.898	9.741				
Rrp41-M1		2.209	14.537	2.206	14.578	2.194	14.524
Rrp41-M44		2.232	13.755	n.d.	n.d.	n.d.	n.d.
Rrp41-M49		1.949	14.518	n.d.	n.d.	n.d.	n.d.
Rrp41-M119	A	2.122	14.784	2.119	14.578	2.116	14.571
	B	2.114	14.679				
Rrp41-M173		1.991	14.258	n.d.	n.d.	n.d.	n.d.
Rrp41-M240		2.050	14.170	n.d.	n.d.	n.d.	n.d.
Rrp41-M266	A	1.855	14.219	n.d.	n.d.	n.d.	n.d.
	B	1.832	14.162				
Rrp41-M281		2.200	14.483	n.d.	n.d.	n.d.	n.d.

[2] Rrp41-I117 and Rrp41-I251 overlap for Exo9. Resonance positions were determined from point mutants.

C

Resonance		monomer		in Exo9		in Exo10	
		^{1}H (ppm)	^{13}C (ppm)	^{1}H (ppm)	^{13}C (ppm)	^{1}H (ppm)	^{13}C (ppm)
Rrp45-I76		0.558	11.335	0.565	11.366	0.557	11.467
Rrp45-I79		0.940	11.756	0.728	11.828	0.809	12.100
Rrp45-I111	A	0.813	7.902	0.936	9.477	0.904	9.624
	B	0.805	7.705				
Rrp45-I133		0.867	8.518	0.807	7.625	0.871	7.583

Rrp45-I152		0.302	10.677	0.240	10.640	0.239	10.626
Rrp45-I168		0.835	11.065	n.d.	n.d.	n.d.	n.d.
Rrp45-I175		0.790	10.966	0.809	11.557	0.734	13.242
Rrp45-I206[3]		0.828	10.716	0.804	10.929	0.800	10.948
Rrp45-I225	A<br>B	1.111	12.501	1.126	13.752	1.113	13.428
Rrp45-I233		n.d.	n.d.	n.d.	n.d.	n.d.	n.d.
Rrp45-I236		n.d.	n.d.	n.d.	n.d.	n.d.	n.d.
Rrp45-I255		1.182	10.954	1.120	10.983	1.122	10.933
	A	2.199	14.608				
Rrp45-M1	B	2.234	14.580	n.d.	n.d.	n.d.	n.d.
	C	2.171	14.766				
Rrp45-M86		2.141	14.477	n.d.	n.d.	n.d.	n.d.
Rrp45-M140		2.063	14.258	n.d.	n.d.	n.d.	n.d.
Rrp45-M227		2.190	14.520	n.d.	n.d.	n.d.	n.d.

[3] Rrp45-I206 overlaps with an unassigned resonance in Exo9 and Exo10.

Table S3: Γ_1 PRE experiments for the Rrp41 entry and exit loop. (**A**) R_1 rates for paramagnetic (para) and diamagnetic (dia) Rrp41^{G71tfmF} (entry loop) Csl4$^{C122S,\ E130C\text{-}TEMPO}$ in Exo9 and Exo9 with RNA as well as paramagnetic relaxation enhancements Γ_1 for these systems. (**B**) R_1 rates for paramagnetic (para) and diamagnetic (dia) Rrp41^{D113tfmF} (exit loop) Rrp42$^{C59S,\ A106C\text{-}TEMPO}$ in Exo9, Exo9 with RNA, Exo10 and Exo10 with RNA as well as paramagnetic relaxation enhancements Γ_1 for these systems. Rates were obtained from Eq. 1. Errors are ±1 SD and were estimated from the fit using Matlab's nlparci function for R_1 or via error propagation for Γ_1.

A

**System**	$R_{1,para}$ **(s^{-1})**	$R_{1,dia}$ **(s^{-1})**	Γ_1 **(s^{-1})**
Rrp41^{G71tfmF} (Csl4$^{C122S,\ E130C\text{-}TEMPO}$)			
Exo9	1.3±0.1	1.0±0.1	0.3±0.2
Exo9 with RNA	1.0±0.1	1.0±0.1	0.0±0.2

B

**System**	$R_{1,para}$ **(s^{-1})**	$R_{1,dia}$ **(s^{-1})**	Γ_1 **(s^{-1})**
Rrp41^{D113tfmF} (Rrp42$^{C59S,\ A106C\text{-}TEMPO}$)			
Exo9	2.5±0.3	1.0±0.1	1.5±0.5
Exo9 with RNA	1.0±0.1	0.9±0.1	0.1±0.2
Exo10	1.4±0.2	1.0±0.1	0.4±0.3
Exo10 with RNA	0.9±0.1	0.9±0.1	0.0±0.2

Table S4: Rrp42-EL fit parameters. Parameters obtained from fitting a two-site exchange model to dynamics and PRE data of Rrp42$^{C59S, A106C-TFA}$ in Exo9 and in Exo10. Errors are ±1 SD and derived from a Monte-Carlo error analysis as described in the method section.

**Parameter**	**Exo9**	**Exo10**
Global		
k_{ex} (s^{-1})	5800±1400	35±35
p_{closed}	0.05±0.01	0.26±0.01
^{19}F Rrp42$^{C59S, A106C-TFA}$		
ω_{open} (ppm)	-84.156 ± 0.002	
ω_{closed} (ppm)	-83.795 ± 0.009	
R_1 (s^{-1})	4.4±0.8	3.6±0.2
R_2 (s^{-1})	100±16	234±9
$R_{2,500}$ (s^{-1})	41.6±0.6	n.d.
$R_{2,600}$ (s^{-1})	53.8±0.8	n.d.
^{19}F Rrp41$^{D113-tfmF}$ (Rrp42$^{C59S, A106C-TEMPO}$)		
$\Gamma_{1,open}$ (s^{-1})	0.54±0.20	0.00±0.00
$\Gamma_{2,open}$ (s^{-1})	111±23	130±26
$\Gamma_{1,closed}$ (s^{-1})	5.3±6.6	4.6±6.7
$\Gamma_{2,closed}$ (s^{-1})	(16.1±0.6)*10^3	(18.8±0.7)*10^3
Solomon-Bloembergen		
r_{open} (Å)	11.6±0.2	
S_{open}^2	0.12±0.02	
$\tau_{i,open}$ (ps)	11±5	
r_{closed} (Å)	6.83±0.08	
S_{closed}^2	0.74±0.04	
$\tau_{i,closed}$ (ns)	24±59	

Table S5: Uniprot accession codes for *Chaetomium thermophilum* proteins used in this study.

Protein	Accession code
Rrp45	G0S755
Rrp41	G0SC21
Rrp43	G0S1P1
Rrp46	G0SCD1
Rrp42	G0RZG4
Mtr3	P0CT46
Rrp40	G0RZX8
Rrp4	G0S9A0
Csl4	G0SE33
Rrp44	G0SEX3

Table S6: Primers and protein constructs used in this study. (**A**) Primers for assignment mutants. (**B**) Primers for constructs other than assignment mutants. (**C**) Protein constructs used for assignments. Note, that assignments of Csl4 Ile-δ1 residues were additionally obtained from conventional assignment methods as described in the method section. (**D**) Internal references for plasmids. (**E**) Protein constructs and experiments conducted on them.

A

Construct	forward primer	reverse primer
Csl4-I18L	GTCAACTGCTGGGTCCGCTGAGTAAATACCAACCGGGTC	GACCCGGTTGGTATTTACTCAGCGGACCCAGCAGTTGAC
Csl4-I60L	GTGAAACGTCTGAATCGCCTGACCCCGGCACCGACG	CGTCGGTGCCGGGGTCAGGCGATTCAGACGTTTCAC
Csl4-I95L	CGGTCGTAAACGCGAACTGCTGCCGGAAGTGGG	CCCACTTCCGGCAGCAGTTCGCGTTTACGACCG
Csl4-I102V	CTGCCGGAAGTGGGTAACGTCGTTCTGTGCCGTGTC	GACACGGCACAGAACGACGTTACCCACTTCCGGCAG
Csl4-I110L	CCGTGTCATCCGCCTGACCCCGCGTCAGG	CCTGACGCGGGGTCAGGCGGATGACACGG
Csl4-I140V	CTGATTCGTGTTCAAGATGTTCGCGCCACCGAAAAAGACC	GGTCTTTTTCGGTGGCGCGAACATCTTGAACACGAATCAG
Csl4-I159L	CGTCCGGGCGATCTGGTGCGCGCCGAAG	CTTCGGCGCGCACCAGATCGCCCGGACG
Csl4-I185L	CAATGAACTGGGCGTTCTGCTGGCGACCAGTGAAG	CTTCACTGGTCGCCAGCAGAACGCCCAGTTCATTG
Csl4-I207L	GAATACCGCGATCCGCTGACCGGCCTGACGGAAC	GTTCCGTCAGGCCGGTCAGCGGATCGCGGTATTC
Csl4-M1A	CTTTATTTTCAGGGCGCCGCGACGACGACGCAACCGAC	GTCGGTTGCGTCGTCGTCGCGGCGCCCTGAAAATAAAG
Csl4-M195A	CAGTGAAGCCGGTAATACGCTGTATCCGGTGTCATGGC	GCCATGACACCGGATACAGCGTATTACCGGCTTCACTG
Rrp41-I30V	GTCGCGTTCATGCCCAAGTTCGCACCCAGG	CCTGGGTGCGAACTTGGGCATGAACGCGAC
Rrp41-I96V	CCGAAGTCGTGGTTTCCGTTGTGATCGCAGG	CCTGCGATCACAACGGAAACCACGACTTCGG
Rrp41-I98V	CGTGGTTTCCATTGTGGTCGCAGGTT	CGGAACTAAAACCTGCGACCACAATGG

	TTAGTTCCG	AAACCACG
Rrp41-I116V	CACGGCCGTAACGATAAACGCGTTATCGAAATGCAAAGC	GCTTTGCATTTCGATAACGCGTTTATCGTTACGGCCGTG
Rrp41-I117V	CCGTAACGATAAACGCATTGTCGAAATGCAAAGCACCG	CGGTGCTTTGCATTTCGACAATGCGTTTATCGTTACGG
Rrp41-I141V	GTTCCCGCATTCACAGGTTACGATCTCGCTGC	GCAGCGAGATCGTAACCTGTGAATGCGGGAAC
Rrp41-I143V	GCATTCACAGATTACGGTCTCGCTGCACGTCC	GGACGTGCAGCGAGACCGTAATCTGTGAATGC
Rrp41-I159V	CTGCTGGCTGCGCTGGTTAATGCGGCAACCCTG	CAGGGTTGCCGCATTAACCAGCGCAGCCAGCAG
Rrp41-I171V	GCTTGTGTTGATGCCGGTGTCCCGATGACCGATTATGTC	GACATAATCGGTCATCGGGACACCGGCATCAACACAAGC
Rrp41-I251V	GTGGACGGCTGTAAACAGGTTCGTGCCATCCTG	CAGGATGGCACGAACCTGTTTACAGCCGTCCAC
Rrp41-I254L	GTAAACAGATTCGTGCCCTGCTGGATCACGTTGTCC	GGACAACGTGATCCAGCAGGGCACGAATCTGTTTAC
Rrp41-I267V	GGTCGTCGCATGGTCCGTGAGGGTGCGGTTG	CAACCGCACCCTCACGGACCATGCGACGACC
Rrp41-M1A	CTTTATTTTCAGGGCGCCGCGCCGCTGGACACGAG	CTCGTGTCCAGCGGCGCGGCGCCCTGAAAATAAAG
Rrp41-M44A	GTAGCTCTTATCTGGAAGCGGGCCATACCAAAGTGATG	CATCACTTTGGTATGGCCCGCTTCCAGATAAGAGCTAC
Rrp41-M49A	GAAATGGGCCATACCAAAGTGGCGTGCGTTGTTACCGGTC	GACCGGTAACAACGCACGCCACTTTGGTATGGCCCATTTC
Rrp41-M119A	CGATAAACGCATTATCGAAGCGCAAAGCACCGTTGCC	GGCAACGGTGCTTTGCGCTTCGATAATGCGTTTATCG
Rrp41-M173A	GTTGATGCCGGTATCCCGGCGACCGATTATGTCGTGG	CCACGACATAATCGGTCGCCGGGATACCGGCATCAAC
Rrp41-M240A	GTTTCTCGCCTGGAAGGCGCGCTGGCGGTCGGTGTG	CACACCGACCGCCAGCGCGCCTTCCAGGCGAGAAAC
Rrp41-M266A	CCAAAAAGGTCGTCGCGCGATCCGTGAGGGTGC	GCACCCTCACGGATCGCGCGACGACCTTTTTGG
Rrp41-M281A	CGTCAGCCTGGATGACGCGGATGAAG	CAATCTTCATCCGCGTCATCCAGGCTG

	ATTG	ACG
Rrp45-I76V	CGTCCGCTGGACGGTGTTTTTACCATCGCAAC	GTTGCGATGGTAAAAACACCGTCCAGCGGACG
Rrp45-I79V	CTGGACGGTATTTTTACCGTGGCAACGGAACTGAGTCCG	CGGACTCAGTTCCGTTGCCACGGTAAAAATACCGTCCAG
Rrp45-I111V	CTGCTGGAAAAAACCGTTCGTCGCAGTGGCGCTC	GAGCGCCACTGCGACGAACGGTTTTTTCCAGCAG
Rrp45-I133V	GGTCAGAAATGTTGGTCAGTTCGCGTTGACGTCCATGTG	CACATGGACGTCAACGCGAACTGACCAACATTTCTGACC
Rrp45-I152V	GACGCGGCCTGCGTGGCAGTGGTTGCAG	CTGCAACCACTGCCACGCAGGCCGCGTC
Rrp45-I168V	CGCAAACCGGATACCAGCGTTGAATCTGGTGTTCTGACC	GGTCAGAACACCAGATTCAACGCTGGTATCCGGTTTGCG
Rrp45-I175V	GAATCTGGTGTTCTGACCGTGTATACGCCGGCCGAAC	GTTCGGCCGGCGTATACACGGTCAGAACACCAGATTC
Rrp45-I206V	GGCGATGAAGGTGAAGTTGCTGTGCTGGACGCG	CGCGTCCAGCACAGCAACTTCACCTTCATCGCC
Rrp45-I225V	CGCGTCGGCTCATGCACGGTGTCGATGAACAAACATG	CATGTTTGTTCATCGACACCGTGCATGAGCCGACGCG
Rrp45-I233V	GATGAACAAACATGGTGAAGTTTGTCAGATTGCAAAACTGGG	CCCAGTTTTGCAATCTGACAAACTTCACCATGTTTGTTCATC
Rrp45-I236V	CATGGTGAAATTTGTCAGGTTGCAAAACTGGGCGGCACC	GGTGCCGCCCAGTTTTGCAACCTGACAAATTTCACCATG
Rrp45-I255L	GCTGCAATGCACCTCTCTGGCTCTGACGAAAG	CTTTCGTCAGAGCCAGAGAGGTGCATTGCAGC
Rrp45-M1A	CTTTATTTTCAGGGCGCCGCGCCGCGTGAAGTG	CACTTCACGCGGCGCGGCGCCCTGAAAATAAAG
Rrp45-M86A	CGGAACTGAGTCCGGCGACCAGCCCGACGTTC	GAACGTCGGGCTGGTCGCCGGACTCAGTTCCG
Rrp45-M140L	CGTTGACGTCCATGTGCTGTCGCACGATGGCAATCTG	CAGATTGCCATCGTGCGACAGCACATGGACGTCAACG
Rrp45-M227A	GGCTCATGCACGATCTCGGCGAACAAACATGGTG	CACCATGTTTGTTCGCCGAGATCGTGCATGAGCC

B

**Construct**	**forward primer**	**reverse primer**
Csl4-C122S	GTTACGATTCTGGTCTCTGGCGATACCGTGCTG	CAGCACGGTATCGCCAGAGACCAGAATCGTAAC
Csl4-E130C	CGTGCTGGACGCGTGCTGGCAGGGTCTGATTC	GAATCAGACCCTGCCAGCACGCGTCCAGCACG
Rrp41-G71amber	GAACCGGGTGCAGGCACCACGTAGGGTGGCGGTGCTG	CAGCACCGCCACCCTACGTGGTGCCTGCACCCGGTTC
Rrp41-Q86amber	GTGGCGGTAGTGGCGGTTAGGGTAAAGAAGCCGAAG	CTTCGGCTTCTTTACCCTAACCGCCACTACCGCCAC
Rrp41-D113amber	GTAAACGCCACGGCCGTAACTAGAAACGCATTATCGAAATG	CATTTCGATAATGCGTTTCTAGTTACGGCCGTGGCGTTTAC
Rrp42-C59S	GTTCTGCACGCGTGTCCTTCGCCGATGGCAC	GTGCCATCGGCGAAGGACACGCGTGCAGAAC
Rrp42-A106C	GACGAAGAAGGCTATTGCAAAGTCGGTGCGGATAAC	GTTATCCGCACCGACTTTGCAATAGCCTTCTTCGTC
Rrp42-Δ93-125	GTTGCGAGCGCCGGCGTCCGCGATG	CATCGCGGACGCCGGCGCTCGCAAC
Rrp45-94ELGESEGESEG95 (Rrp45-L)	CGAACTGGGCGAATCTGAAGGCGAATCTGAAGGCCGTCCGACCGAAACGGAAGTTC	GAACTTCCGTTTCGGTCGGACGGCCTTCAGATTCGCCTTCAGATTCGCCCAGTTCG
Rrp44-PIN	GCATTTCGTTCGGGCGTTATTCTTTAATCGTTTCTTC	GCATTTCGTTCGGGCGTTATTCTTTAATCGTTTCTTC
Rrp44-PIN+CS	CGTGCTGGATTGCCTGCCGAAAACGGGCTAACACGACTGGCGCGTGCCGGAAG	CTTCCGGCACGCGCCAGTCGTGTTAGCCCGTTTTCGGCAGGCAATCCAGCACG
Rrp44-D168N	CTGTGAACGATCGTAATAACCGCGCCGTTCG	CGAACGGCGCGGTTATTACGATCGTTCACAG
Rrp44-D536N	GGTTGTCAGGACATTAATGATGCACTGCACAGTCG	CGACTGTGCAGTGCATCATTAATGTCCTGACAACC

C

**Construct**	**Internal database reference**	**Methyl-TROSY HMQC acquired**		
		**monomer**	**Exo9**	**Exo10**
Csl4-I60L	#1972	yes	no	no
Csl4-I95L	#1970	yes	no	no
Csl4-I102V	#2160	no	yes	no
Csl4-I110L	#2139	yes	yes	no
Csl4-I159L	#2141	yes	yes	no
Csl4-I185L	#2140	yes	no	no
Csl4-I207L	#2138	yes	no	no
Csl4-M1A	#1969	yes	no	no
Csl4-M195A	#1971	yes	no	no
Rrp41-I30V	#2399	yes	no	no
Rrp41-I96V	#2385	yes	no	no
Rrp41-I98V	#2380	yes	no	no
Rrp41-I116V	#2381	yes	yes	no
Rrp41-I117V	#2387	yes	yes	yes
Rrp41-I141V	#2401	yes	yes	yes
Rrp41-I143V	#2402	yes	yes	no
Rrp41-I159V	#2403	yes	yes	yes
Rrp41-I171V	#2404	yes	yes	no
Rrp41-I251V	#2400	yes	yes	yes
Rrp41-I254L	#2382	yes	no	no
Rrp41-I267V	#2424	yes	yes	no
Rrp41-M1A	#2386	yes	no	no
Rrp41-M44A	#2379	yes	no	no
Rrp41-M49A	#2405	yes	no	no
Rrp41-M119A	#2384	yes	no	no
Rrp41-M173A	#2406	yes	no	no
Rrp41-M240A	#2407	yes	no	no
Rrp41-M266A	#2408	yes	no	no
Rrp41-M281A	#2409	yes	no	no
Rrp45-I76V	#2195	yes	no	no
Rrp45-I79V	#2196	yes	yes	yes

Rrp45-I111V	#2197	yes	yes	yes
Rrp45-I133V	#2191	yes	yes	yes
Rrp45-I152V	#2192	yes	no	no
Rrp45-I168V	#2193	yes	yes	no
Rrp45-I175V	#2194	yes	yes	yes
Rrp45-I206V	#2202	yes	yes	no
Rrp45-I225V	#2203	yes	no	no
Rrp45-I233V	#2198	yes	no	no
Rrp45-I236V	#2204	yes	no	no
Rrp45-I255L	#2205	yes	no	no
Rrp45-M1A	#2199	yes	no	no
Rrp45-M86A	#2200	yes	no	no
Rrp45-M140L[1]	#2253	no	no	no
Rrp45-M227A	#2201	yes	no	no

[1] Construct aggregated. Assignment possible via exclusion since all other Met were assigned.

D

**Construct**	**Internal database reference**
Csl4	#1247
Rrp4	#1233
Rrp40	#1213
Rrp41	#1245
Rrp42	#1214
Rrp44	#1242
Rrp45	#1215
Mtr3	#1246
Rrp41-Rrp45	#1351
Mtr3-Rrp42	#1355
Rrp43-Rrp46	#1353
$Csl4^{C122S, E130C}$	#2741
$Rrp42^{C59S}$	#2172
$Rrp42^{C59S, A106C}$	#2176
$Rrp44^{D168N, D536N}$	#1671
$Rrp44^{PIN}$ (residues 1-244 of FL Rrp44)	#1835
$Rrp44^{PIN+CS}$ (residues 1-499 of FL Rrp44)[2]	#2099

Mtr3-Rrp42$^{\Delta 93\text{-}125}$	#2445
Rrp45-94ELGESEGESEG95 (Rrp45-L)	#2539
Rrp41$^{\mathrm{G71amber}}$	#2716
Rrp41$^{\mathrm{Q86amber}}$	#2661
Rrp41$^{\mathrm{D113amber}}$	#2664

[2]contained an artifact E498T

E

**Construct**	**Type(s) of experiment**
Exo9	Cryo-EM, X-ray crystallography
U-^{15}N/^{13}C Csl4	HNCACB, HNCA, HNCOCACB, HNCO, HNCACO, H(CCCO)NH, C(CCO)NH
Monomeric IM-labeled Csl4	Methyl TROSY HMQC
Monomeric IM-labeled Rrp41	Methyl TROSY HMQC
Monomeric IM-labeled Rrp45	Methyl TROSY HMQC
Exo9, IM-labeled Csl4	Methyl TROSY HMQC
Exo9, IM-labeled Rrp41	Methyl TROSY HMQC
Exo9, IM-labeled Rrp45	Methyl TROSY HMQC
Exo10, IM-labeled Csl4	Methyl TROSY HMQC
Exo10, IM-labeled Rrp41	Methyl TROSY HMQC
Exo10, IM-labeled Rrp45	Methyl TROSY HMQC
Exo9, 46mer RNA, IM-labeled Csl4	Methyl TROSY HMQC
Exo9, 46mer RNA, IM-labeled Rrp41	Methyl TROSY HMQC
Exo9, 46mer RNA, IM-labeled Rrp45	Methyl TROSY HMQC
Exo10, Rrp44$^{\mathrm{D168N,\ D536N}}$, 46mer RNA, IM-labeled Csl4	Methyl TROSY HMQC
Exo10, Rrp44$^{\mathrm{D168N,\ D536N}}$, 46mer RNA, IM-labeled Rrp41	Methyl TROSY HMQC
Exo10, Rrp44$^{\mathrm{D168N,\ D536N}}$, 46mer RNA, IM-labeled Rrp45	Methyl TROSY HMQC
Exo9, Rrp42$^{\mathrm{C59S,\ A106C\text{-}TEMPO}}$, IM-labeled Csl4	Methyl PRE
Exo9, Rrp42$^{\mathrm{C59S,\ A106C\text{-}TEMPO}}$, IM-labeled Rrp41	Methyl PRE
Exo10, Rrp42$^{\mathrm{C59S,\ A106C\text{-}TEMPO}}$, IM-labeled Rrp41	Methyl PRE
Exo9, Rrp42$^{\mathrm{C59S,\ A106C\text{-}TEMPO}}$, IM-labeled Rrp45	Methyl PRE
Exo10, Rrp42$^{\mathrm{C59S,\ A106C\text{-}TEMPO}}$, IM-labeled Rrp45	Methyl PRE
Exo9, Rrp42$^{\mathrm{C59S,\ A106C\text{-}TEMPO}}$, 46mer RNA, IM-labeled Rrp45	Methyl PRE
Exo10, Rrp44$^{\mathrm{D168N,\ D536N}}$, Rrp42$^{\mathrm{C59S,\ A106C\text{-}TEMPO}}$, 46mer RNA, IM-labeled Rrp45	Methyl PRE

Exo9, Rrp41^{Q86tfmF}	^{19}F 1D, CPMG RD
Exo10, Rrp41^{Q86tfmF}	^{19}F 1D, CPMG RD
Exo10, Rrp44$^{D168N, D536N}$, 46mer RNA, Rrp41^{Q86tfmF}	^{19}F 1D
Exo9, Csl4$^{C122S, E130C-TEMPO}$, Rrp41^{G71tfmF}	^{19}F Γ_1 PRE, ^{19}F Γ_2 PRE
Exo9, Csl4$^{C122S, E130C-TEMPO}$, 46mer RNA, Rrp41^{G71tfmF}	^{19}F Γ_1 PRE, ^{19}F Γ_2 PRE
Exo9, Rrp41^{D113tfmF}	^{19}F 1D, ^{19}F CPMG RD
Exo10, Rrp41^{D113tfmF}	^{19}F 1D, ^{19}F CPMG RD
Exo10, Rrp44$^{D168N, D536N}$, 46mer RNA, Rrp41^{D113tfmF}	^{19}F 1D
Exo9, Rrp42$^{C59S, A106C-TEMPO}$, Rrp41^{D113tfmF}	^{19}F Γ_1 PRE, ^{19}F Γ_2 PRE
Exo9, Rrp42$^{C59S, A106C-TEMPO}$, 46mer RNA, Rrp41^{D113tfmF}	^{19}F Γ_1 PRE, ^{19}F Γ_2 PRE
Exo10, Rrp42$^{C59S, A106C-TEMPO}$,Rrp41^{D113tfmF}	^{19}F Γ_1 PRE, ^{19}F Γ_2 PRE
Exo10, Rrp44$^{D168N, D536N}$, Rrp42$^{C59S, A106C-TEMPO}$, 46mer RNA, Rrp41^{D113tfmF}	^{19}F Γ_1 PRE, ^{19}F Γ_2 PRE
wtRrp42^{TFA}	^{19}F 1D
Rrp42$^{C59S, TFA}$	^{19}F 1D
Rrp42$^{C59S, A106C-TFA}$	^{19}F 1D
Mtr3, Rrp42$^{C59S, A106C-TFA}$	^{19}F 1D
Exo9, Rrp42$^{C59S, A106C-TFA}$	^{19}F 1D, ^{19}F CEST, ^{19}F CPMG RD
Exo9, 46mer RNA, Rrp42$^{C59S, A106C-TFA}$	^{19}F 1D, ^{19}F CEST, ^{19}F CPMG RD
Exo10, Rrp42$^{C59S, A106C-TFA}$	^{19}F 1D, ^{19}F CEST, ^{19}F/^{19}F EXSY
Exo10, Rrp44$^{D168N, D536N}$, 46mer RNA, Rrp42$^{C59S, A106C-TFA}$	^{19}F 1D, ^{19}F CEST, ^{19}F CPMG RD
Exo10, 80mer RNA, Rrp42$^{C59S, A106C-TFA}$	activity assay
Exo10, Rrp44^{PIN}, Rrp42$^{C59S, A106C-TFA}$	^{19}F 1D, ^{19}F CPMG RD
Exo10, Rrp44^{PIN+CS}, Rrp42$^{C59S, A106C-TFA}$	^{19}F 1D, ^{19}F CPMG RD
Exo10, 80mer RNA	activity assay
Exo10, Rrp42$^{\Delta 93-125}$, 80mer RNA	activity assay
Exo10, Rrp45-L, 80mer RNA	activity assay
Exo10, Rrp45-L, Rrp42$^{\Delta 93-125}$, 80mer RNA	activity assay

Table S7: Sequences of RNAs used in this study.

RNA	Sequence	Internal database reference
46mer	GGAGGAGAGGUGAGGAGAGAGGAGAGGAAGGAAGGGAAGAAAGAAG	#72
80mer	GGAAGGAGAGGAAGGAAAGGUGGGAAGAGGAAGGAGAGGAGGGAAG AAAGAAGAGGAGAGGAAGGAAGGGAAGAAAGAAG	#39

Table S8: Parameters for CPMG relaxation dispersion experiments.

**System**	ν_{CPMG}		**number of**	T_{CPMG} **(ms)**
	**min (Hz)**	**max (Hz)**	**frequencies**	
Exo9 Rrp42$^{C59S,\ A106C\text{-}TFA}$	100	5000	25	20
Exo10 PIN Rrp42$^{C59S,\ A106C\text{-}TFA}$	250	5000	20	8
Exo10 PIN+CS Rrp42$^{C59S,\ A106C\text{-}TFA}$	250	5000	20	8
Exo9 Rrp42$^{C59S,\ A106C\text{-}TFA}$ + RNA	100	5000	25	20
Exo10 Rrp42$^{C59S,\ A106C\text{-}TFA}$ + RNA	100	5000	25	20
Exo9 Rrp41^{D113tfmF}	500	5000	10	4
Exo10 Rrp41^{D113tfmF}	500	5000	10	2
Exo9 Rrp41^{Q86tfmF}	167	5000	20	6
Exo10 Rrp41^{Q86tfmF}	167	5000	20	6

Table S9: Elution program for HPLC runs of activity assay samples.

Time interval (min)	Linear changes in concentration of buffer F (%)
0 – 3	0
3 – 4	0 – 20
4 – 11	20 – 50
11 – 12	50 – 100
12 – 14	100
14 – 19	0

Table S10: Expanded analysis of exosome activity. (**A**) Time points considered in various analyses of exosome activity data. (**B**) Catalytic rates, k_{cat}, obtained for various analyses of exosome activity data. Rates were obtained from three biological replicates with three technical repeats. Errors are ±1 SD. See Fig. 6 in the main text for analysis 1 and fig. S26 for analyses 2 – 4. wt = wtExo10, Δ = Exo10 Rrp42$^{\Delta 93\text{-}125}$, L = Exo10 Rrp45-L, L-Δ = Exo10 Rrp45-L Rrp42$^{\Delta 93\text{-}125}$.

A

**Analysis**	**Included time points (min)**			
	**wt**	**Δ**	**L**	**L-Δ**
1	0-3	0-3	0-10	0-10
2	0-3	0-3	0-10	0-5
3	0-5	0-5	0-10	0-10
4	0-10	0-10	0-10	0-10

B

**Analysis**	k_{cat} **(nt/min)**			
	**wt**	**Δ**	**L**	**L-Δ**
1	87±29	87±37	3.8±2.3	11±2.8
2	87±29	87±37	3.8±2.3	13±4.8
3	68±23	75±39	3.8±2.3	11±2.8
4	42±16	51±23	3.8±2.3	11±2.8

Supplementary References

1. Y. Z. Tan, P. R. Baldwin, J. H. Davis, J. R. Williamson, C. S. Potter, B. Carragher, D. Lyumkis, Addressing preferred specimen orientation in single-particle cryo-EM through tilting. *Nat. Methods* **14**, 793–796 (2017).

2. P. Gerlach, J. M. Schuller, F. Bonneau, J. Basquin, P. Reichelt, S. Falk, E. Conti, Distinct and evolutionary conserved structural features of the human nuclear exosome complex. *Elife* **7**, e38686 (2018).

3. E. Lorentzen, A. Dziembowski, D. Lindner, B. Seraphin, E. Conti, RNA channelling by the archaeal exosome. *EMBO Rep.* **8**, 470–476 (2007).

4. F. Bonneau, J. Basquin, J. Ebert, E. Lorentzen, E. Conti, The yeast exosome functions as a macromolecular cage to channel RNA substrates for degradation. *Cell* **139**, 547–559 (2009).

5. A. Rangadurai, H. Shi, H. M. Al-Hashimi, Extending the sensitivity of CEST NMR spectroscopy to micro-to-millisecond dynamics in nucleic acids using high-power radio-frequency fields. *Angew. Chem., Int. Ed.* **59**, 11262–11266 (2020).

6. D. L. Makino, M. Baumgärtner, E. Conti, Crystal structure of an RNA-bound 11-subunit eukaryotic exosome complex. *Nature* **495**, 70–75 (2013).

Figure S3c – uncropped gel

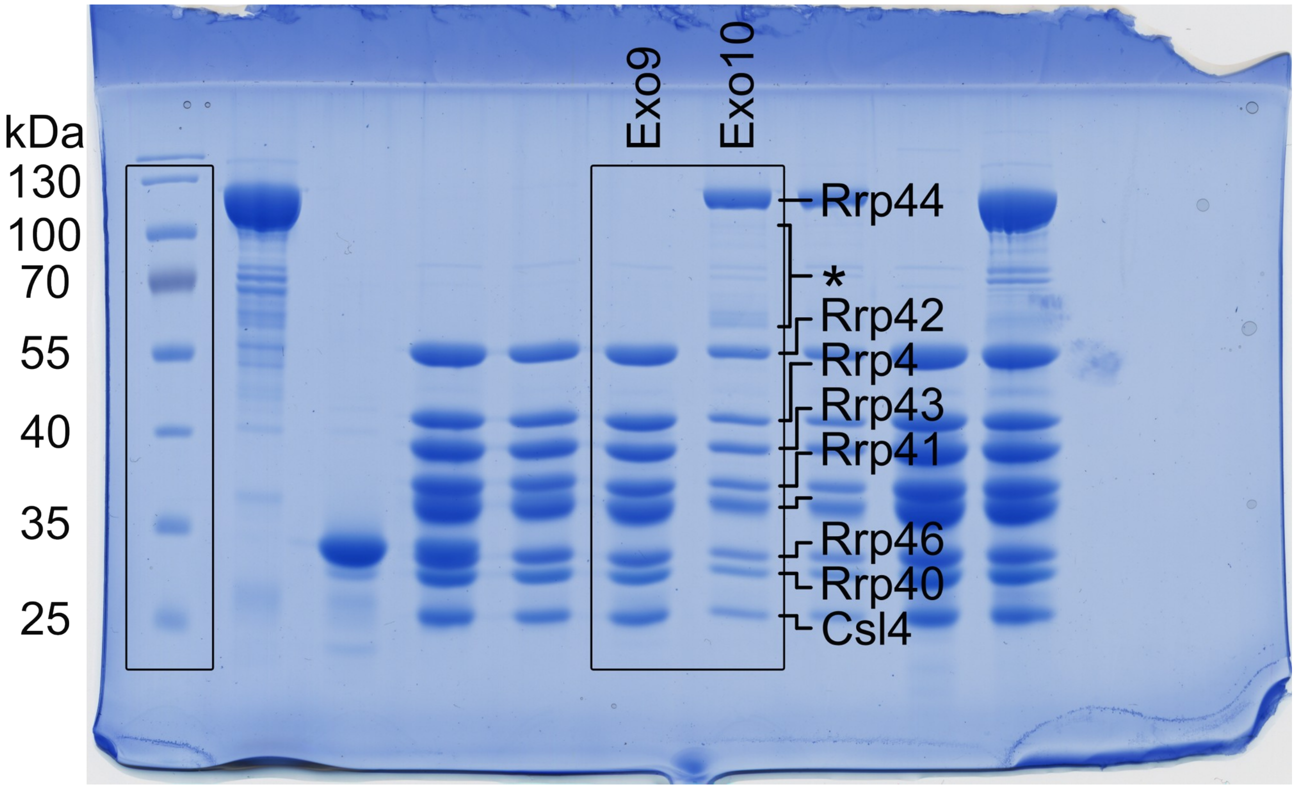

Figure S14 – uncropped gels

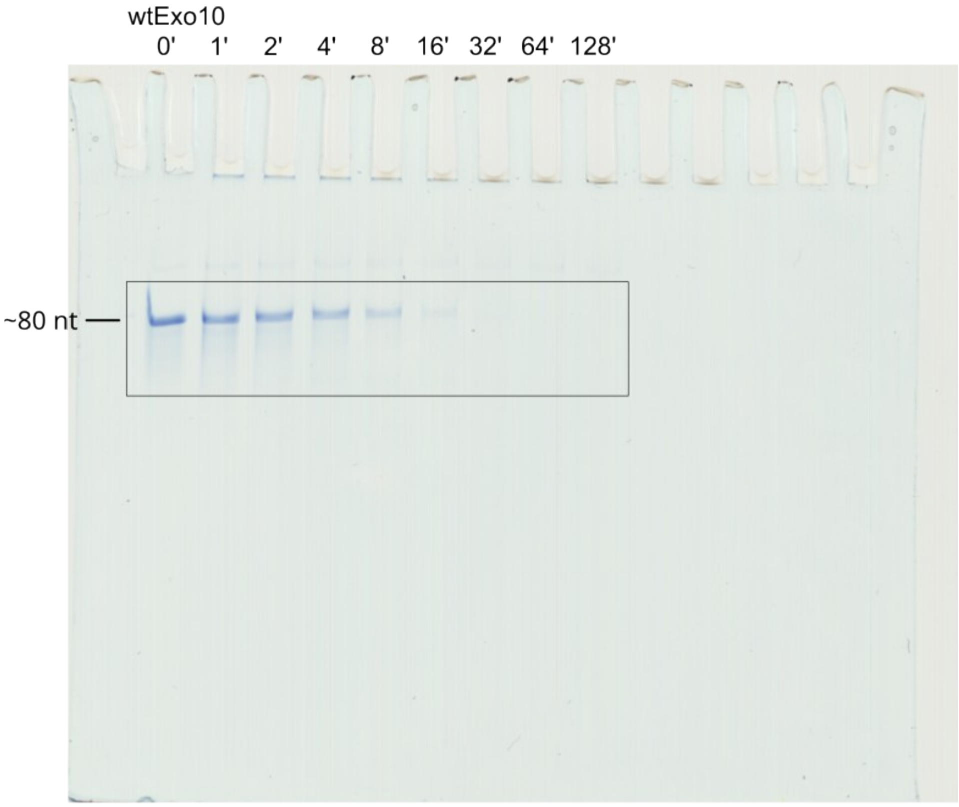

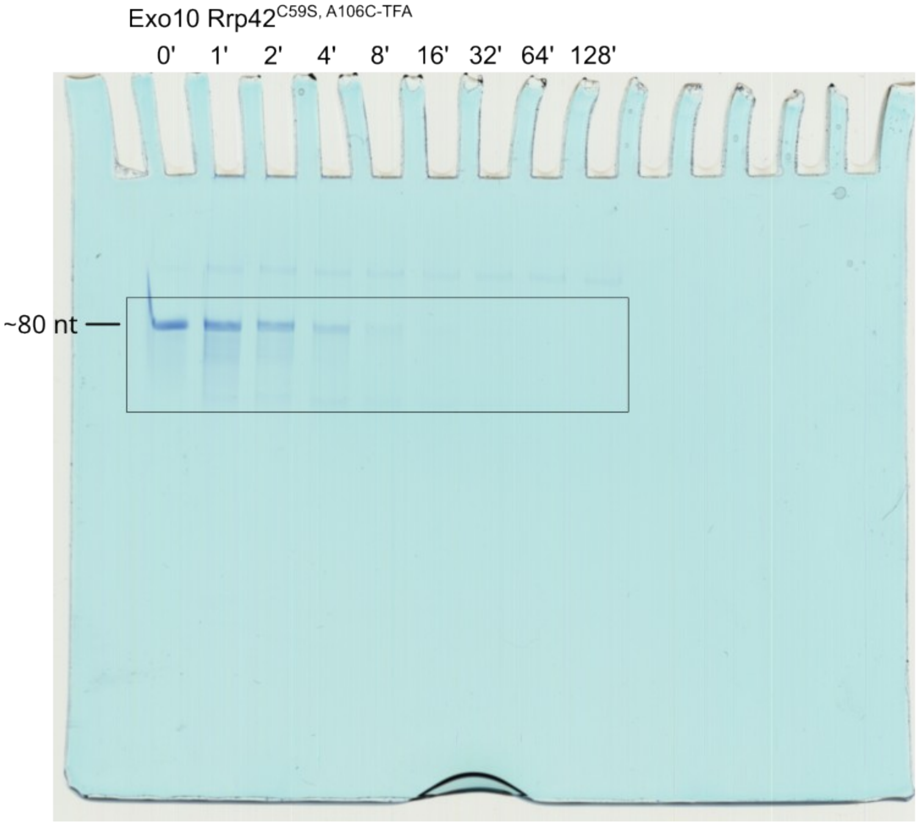

Supplement: Supplementary file 1 — Supplementary Information [file 41467_2025_62982_MOESM1_ESM.pdf]
