# Supplementary material for: 4D structural biology–quantitative dynamics in the eukaryotic RNA exosome complex
Source: Nat Commun. 2025 Aug 24;16:7896. doi: 10.1038/s41467-025-62982-6 (PMC12375074; doi:10.1038/s41467-025-62982-6)
Supplement: Supplementary file 2 — Description of Additional Supplementary Files [file 41467_2025_62982_MOESM2_ESM.pdf]

Description of Additional Supplementary Files

Supplementary Movie 1: Morph between the open and closed state of Rrp42-EL (dark green) for a representative structure (see Supplementary Figure 21) of the MD simulations. Rrp42 is in green, Rrp41 is in red, Rrp45 is in orange and Csl4 is in blue.
